# COVID-19 Severity and Mortality in Two Pandemic Waves in Poland and Predictors of Poor Outcomes of SARS-CoV-2 Infection in Hospitalized Young Adults

**DOI:** 10.3390/v14081700

**Published:** 2022-07-31

**Authors:** Laura Ziuzia-Januszewska, Marcin Januszewski, Joanna Sosnowska-Nowak, Mariusz Janiszewski, Paweł Dobrzyński, Alicja A. Jakimiuk, Artur J. Jakimiuk

**Affiliations:** 1 Department of Otolaryngology, Central Clinical Hospital of the Ministry of Interior and Administration, 02-507 Warsaw, Poland; joanna.sosnowska@cskmswia.gov.pl (J.S.-N.); mariusz.janiszewski@cskmswia.gov.pl (M.J.); pawel.dobrzynski@cskmswia.gov.pl (P.D.); 2 Department of Obstetrics and Gynecology, Central Clinical Hospital of the Ministry of Interior and Administration, 02-507 Warsaw, Poland; lek.med.mjanuszewski@gmail.com (M.J.); jakimiuk@yahoo.com (A.J.J.); 3 Department of Plastic Surgery, Central Clinical Hospital of the Ministry of Interior and Administration, 02-507 Warsaw, Poland; alajakimiuk@hotmail.com; 4 Center for Reproductive Health, Institute of Mother and Child, 01-211 Warsaw, Poland

**Keywords:** COVID-19, SARS-CoV-2, alpha variant, severity, mortality, predictors, young adults

## Abstract

SARS-CoV-2 variants pose a significant threat to global public health. However, their influence on disease severity, especially among young adults who may exhibit different clinical characteristics, is debatable. In this retrospective study of 229 young adults hospitalized with COVID-19, we investigated the differences between Poland’s second and third waves of the pandemic. To identify potential predictors of severe COVID-19 in young adults, we analyzed patient characteristics and laboratory findings between survivors and non-survivors and we performed logistic regression to assess the risk of death, mechanical ventilation, and intensive care unit treatment. We found no increase in COVID-19 severity comparing the third and second waves of the pandemic, indicating that the alpha variant had no influence on disease severity. In addition, we found that factors, such as obesity, comorbidities, lung involvement, leukocytosis, neutrophilia, lymphopenia, higher IG count, the neutrophil-to-lymphocyte ratio, C-reactive protein, procalcitonin, interleukin-6, D-Dimer, lactate dehydrogenase, high-sensitive troponin I, creatine kinase-myocardial band, myoglobin, N-terminal-pro-B-type natriuretic peptide, creatinine, urea and gamma-glutamyl transferase, lower estimated glomerular filtration rate, albumin, calcium and vitamin D3, possibly a decrease in red blood cell counts, hemoglobin and hematocrit, and an increase in creatine kinase during hospitalization may be associated with poor outcomes of COVID-19.

## 1. Introduction

Coronavirus disease 2019 (COVID-19), declared a pandemic by the World Health Organization (WHO) on 11 March 2020 [1], is caused by severe acute respiratory syndrome coronavirus-2 (SARS-CoV-2).

SARS-CoV-2 is a highly infectious RNA virus, which utilizes its spike (S) protein for cellular entry by binding to the host angiotensin-converting enzyme 2 (ACE-2) receptor [2]. This interaction requires the cleavage of the S protein by cell proteases, including transmembrane protease serine 2 (TMPRSS2) [3].

As of 30 June 2022, 544,324,069 cases of COVID-19 had been reported worldwide, causing 6,332,963 deaths [4]. In Poland, despite there being low incidence and mortality rates during the first European wave of the pandemic during the spring of 2020, the second and the third waves were both associated with high case and mortality rates, with the numbers of daily positive SARS-CoV-2 laboratory test results peaking on 7 November 2020 at 27,875 cases and then again on 1 April 1 2021, at 35,251 cases. [5].

By the end of 2020, the emergence of the new variants of SARS-CoV-2, associated with changes in viral transmissibility, clinical presentation, and/or effectiveness of preventative, diagnostic, and therapeutic measures, posed a further threat to global public health [6]. This has prompted researchers and health organizations to characterize the variants of concern (VOCs), including alpha (B.1.1.7), beta (B.1.351), and later gamma (P.1), delta (B.1.617.2), and omicron (B.1.1.529) variants [6,7]. Notably, only omicron is still considered a currently circulating VOC, while the remaining previous VOCs are now labeled by the WHO as “previously circulating VOCs” [6], by the CDC as “variants being monitored” (VBMs) [7], and by the ECDC as “de-escalated variants” [8].

The alpha variant (B.1.1.7), first identified in the UK and then spreading worldwide, was defined by multiple mutations, including changes in the spike protein (N501Y, A570D, D614G, P681H, T716I, S982A, D1118H, del 69–70, del 144), with case rates increasing from fewer than 5% of all SARS-CoV-2 infections to more than 60% between November and mid-December 2020, causing a sharp increase in COVID-19 incidence, hospitalization, and mortality [9]. In Poland, according to the ECDC report of 15 February 2021, incidence of the alpha variant among all cases sequenced in the preceding weeks was at 9% [8], while according to ECDC data on SARS-CoV-2 variants in the EU/EEA [10] only one case of B.1.1.7 was detected until the end of 2020 (in the week 2020-52), and from the beginning to the end of January 2021 the incidence of alpha variant was 9.5% (52 reported detections of B.1.1.7 variant out of 548 sequences carried out), with the overall incidence in weeks 2020-37 to 2021-04, i.e., from 7 September 2020 to 31 January 2021, of 6.5% (53/821), and then gradually increasing, exceeding 50% in the week 2021-07 (15 February to 21 February 2021) and rapidly rising further to over 90% in March, 2021, with the overall incidence in weeks 2021-06 to 2021-23 (8 February to 13 June 2021) of 92.4% (15,442/16,709) [10]. These data indicate that the third wave of the COVID-19 pandemic in Poland was mainly caused by the alpha variant, while the second wave was caused by the previously known SARS-CoV-2 variants. Hence, one can suspect that the differences between these waves may indicate the differences between the disease courses depending on the causative variants.

Several studies suggested the alpha variant was more transmissible than the previously identified SARS-CoV-2 variants [11,12], and this was hypothesized as resulting from the higher viral load and longer detectability in respiratory secretions, possibly attributable to mutations of the spike protein, including in the receptor-binding domain and adjacent to the furin-cleavage site, and therefore affecting viral cell entry [11,13]. Some reports indicated greater disease severity associated with the alpha variant [14,15,16], however, others did not find this relationship [13]. Similarly, the widely debated possibility of increased severity among young people remains unclear [17,18]. Interestingly, Kayano et al. [19] reported higher odds of severe illness and death in patients infected with alpha variant compared to preexisting strains, but these results were statistically insignificant among patients aged <40 years and >79 years old with respect to severe cases and among patients aged <50 years with respect to deaths. Although the later delta variant is generally considered to be associated with greater disease severity, data are also inconsistent [20,21,22,23]. Nevertheless, the alpha, beta, gamma and delta variants have been de-escalated from being VOCs, as no longer considered to pose significant risk to public health [24]. Currently most predominant omicron variant, still labeled as VOC, appears to cause less severe disease [8,25,26], however, still more data are needed [27].

Although older age is a risk factor for both the incidence and worse prognosis of COVID-19 [28,29,30,31,32], severe disease and death have also been observed among young adults [31,33,34]. Moreover, clinical characteristics and laboratory test results in younger people seem to be different from those in elderly patients [31,34], and this may indicate a different pathogenesis of COVID-19 in these age groups [34]. Furthermore, although work, education, and other social settings put young people at a higher risk of SARS-CoV-2 exposure, this age group appears to be less likely to comply with preventative measures [35]. However, there is limited data available in the literature on the predictors of the severe course of this disease in young adults [31,32,33,34].

The primary aim of this study was to investigate the differences between the course of disease in young adult inpatients comparing the second and the third waves of the COVID-19 pandemic in Poland, possibly reflecting differences in the severity of COVID-19 in hospitalized young adults depending on the causative SARS-CoV-2 variant (alpha vs. wild-type variants). The secondary aim was to identify the potential predictors of severe course of COVID-19 in this age group.

## 2. Materials and Methods

### 2.1. Subjects and Settings

This single-centered, retrospective study of hospitalized young adults with COVID-19 during the second and the third wave of pandemic in Poland was conducted at the Central Clinical Hospital of the Ministry of the Interior and Administration in Warsaw, Poland, which was designated by the government for the treatment of patients suffering from COVID-19.

The inclusion criteria were adults not younger than 18 and not older than 45 years, with laboratory-confirmed SARS-CoV-2 infection, who were admitted due to severe COVID-19 (i.e., meeting hospital admission criteria for COVID-19, with oxygen saturation of 94% or less on room air or the need for oxygen therapy [36]) during the second or third waves of the COVID-19 pandemic in Poland. SARS-CoV-2 infection was diagnosed by either RT-PCR or rapid antigen test performed on nasopharyngeal samples, following the protocols supplied by the testing kits’ manufacturers (GeneProof SARS-CoV-2 PCR Kit, GeneProof a.s., Brno, Czech Republic; GeneFinder TM COVID-19 Plus RealAmp Kit; Panbio™ COVID-19 AG Rapid Test Device, Abbott, Abbot Rapid Diagnostics Jena GmbH, Jena, Germany; Bioeasy 2019-nCoV Ag Fluorescence, Shenzhen Bioeasy Biotechnology Co. Ltd., Shenzhen, China), all of which have approximately 100% specificity.

Patients admitted for reasons other than COVID-19 (e.g., trauma, other acute conditions, or serious deterioration in the course of chronic diseases) were excluded from the study. Pregnant women were also excluded due to the possible influence of pregnancy on the severity of the disease [37] and on the results of potential laboratory predictors [38].

In our study, we defined the beginning of a wave as the first day of an at least 14-day period as a continuous increase in the 7-day average number of new cases. The end of a wave was defined as the last day of an at least 14-day period with a continuous decrease in the 7-day average number of new cases, preceding the start of a new wave. Therefore, the second wave was defined as the period from 12 September 2020 to 27 January 2021, and the third wave from 11 February 2021 to 10 June 2021 [5]. It should be noted that, according to the ECDC data on SARS-CoV-2 variants in the EU/EEA, the incidence of the alpha variant in Poland from 7 September 2020 to 31 January 2021 was 6.5%, while in the period from 8 February to 13 June 2021, the alpha variant constituted 92.4% of identified strains [10].

### 2.2. Clinical Outcomes

Data regarding the patient characteristics (including age, sex, smoking status, and comorbidities), the course of the disease (including the need for Intensive Care Unit (ICU) admission, respiratory support, vasopressors, continuous renal replacement therapy (CRRT), complications, and outcome), and laboratory results, imaging results and blood oxygen saturation (SpO_2_) were transcribed from electronic medical records and entered into the database after anonymization. The criteria for ICU admission were acute respiratory distress syndrome (ARDS), the need for mechanical ventilation (MV), symptoms of shock or multi-organ failure and impaired consciousness. The laboratory parameters were collected from two time points: at admission to hospital (+/−2 days, “at admission”) and on the 7th day of hospitalization (+/−2 days, “7th DOH”). The patients’ characteristics in the second and the third waves were compared. Moreover, to assess the role of potential predictors of disease severity, we used in-hospital death as the main outcome measure by comparing the general and clinical characteristics of the survivors and non-survivors. Additionally, to assess the differences in disease severity, we analyzed the correlations between the patients’ characteristics and the risk for MV and ICU treatment, and performed logistic regression to identify factors associated with the risk of death, MV, and ICU treatment.

### 2.3. Ethical Concerns

The study was approved by the Ethics Committee of the Central Clinical Hospital of the Ministry of the Interior and Administration in Warsaw (decision number 110/2021, date of approval 24 August 2021) with a waiver for written informed consent due to the retrospective nature of the study and the data anonymization. The study was performed in accordance with the ethical standards of the Declaration of Helsinki and its later amendments.

### 2.4. Statistical Analysis

As most variables were non-normally distributed, continuous variables were reported as median and interquartile range (IQR) and compared with a Mann–Whitney U-test, while categorical variables were presented as the number of patients and percentages and compared with the chi-squared test or Fisher’s exact test as appropriate. For some of the quantitative variables both raw data and the groups of ranges were analyzed with the appropriate tests. The correlations between the variables and the need for MV and the ICU treatment were examined using the Spearman correlation analysis for continuous variables, and associations of the nominal variables were investigated with the chi-squared test. These analyses were performed with R software (version 4.0.4; R foundation for statistical computing, Vienna, Austria). A two-sided *p*-value < 0.05 was considered statistically significant. As there was no adjustment for multiple comparison, these findings should be considered as exploratory. In cases where outliers were identified, the data were also analyzed after their elimination and replacement with means to assess their impact on the results. If this led to a different result, these data are also presented.

Logistic regression analysis was performed using *Statistica* software (version 13.3; StatSoft, Poland) to determine the association of patients’ characteristics and laboratory parameters and the risk of death, ICU admission, and mechanical ventilation. Non-linear data were categorized. Variables with more than 20% of missing values were not considered in this analysis, and in other cases, missing values were imputed using the Weight of Evidence tool. Univariate analysis was first performed, and significant variables obtained on admission that were significant in the univariate analysis were further included in stepwise multivariate logistic regression. ROC (receiver operating characteristic) curve analysis was conducted to assess the predictive ability of covariates and models in multivariate logistic regression. Graphical presentation of data was carried out using *Statistica* software (version 13.3).

## 3. Results

### 3.1. General Characteristics and Comparison of the Second and Third Waves

Briefly, 229 COVID-19 patients were included in our study (172 men and 57 women), of which 75 patients were attributed to the second wave (59 men and 16 women), and 154 to the third wave (113 men and 41 women) of the COVID-19 pandemic. There was no significant difference regarding gender distribution between the two analyzed waves (*p* = 0.480). The median age in both groups was 40 years (IQR 33.5–42 years in the second wave and 35–43 years in the third wave, range 20–45 years in both waves), with no statistically significant difference between the waves. None of the patients was vaccinated against COVID-19 nor did they have documented previous SARS-CoV-2 infection. The comparison of the general and clinical patients’ characteristics in the two analyzed waves is summarized in Table 1, Table 2 and Table S1.

There were no significant differences between the second and the third wave regarding weight and BMI. It is worth noting that normal BMI (below 25 kg/m^2^) was found in only 12.34% of patients (*n* = 19). There were also no significant differences regarding patients’ smoking status.

Ninety-three patients (40.61%) had at least one comorbidity, comprising 35 (46.67%) and 58 (37.66%) patients from the second and third waves, respectively, and this difference was not statistically significant. The most common comorbidities were hypertension (14.85%), asthma (7.86%), and diabetes (7.86%). There were no statistically significant differences regarding any of the reported comorbidities between the two waves.

The most commonly reported COVID-19 symptoms were dyspnea (91.7%), fever (85.15%), cough (85.15%), and fatigue (56.77%). Other symptoms included myalgia (29.26%), smell or taste disorders (18.78%), headache (18.34%), diarrhea (17.9%), nausea and/or emesis (11.79%), sore throat (7.86%), and hemoptysis (3.49%). There were no statistically significant differences regarding any of the reported symptoms between the two waves.

The median percentage of lung involvement on computed tomography (CT) was 31%, comprised of 30% in the second wave and 33% in the third wave (*p* = 0.319). Lung involvement of at least 50% was found in 24.64% of the patients, comprising 20.31% in the second wave and 26.57% in the third wave (*p* = 0.429).

The median period from the onset of symptoms to hospital admission was eight days (IQR 6–10 days) in the second wave and nine days (IQR 7–11 days) in the third wave (*p* = 0.074). The median duration of hospitalization among survivors was significantly longer (*p* = 0.036) in the second wave (11 days, IQR 9–16 days) compared to the third wave (10 days, IQR 8–13.25 days).

Regarding medical treatment, 227 patients (99.1%, including 100% in the second wave and 98.7% in the third wave) received low molecular-weight heparin (LMWH), 222 patients (96.9%, including 98.7% in the second wave and 96.1% in the third wave) received dexamethasone, 166 patients (72.5%, including 74.7% in the second wave and 71.4% in the third wave) received antibiotics, 59 patients (25.8%, including 25.3% in the second wave and 26% in the third wave) received remdesivir, nine patients (3.9%, including 3.9% in the second wave and 4% in the third wave) received tocilizumab, and 20 patients (8.7%, including 9.3% in the second wave and 8.4% in the third wave) received convalescent plasma, with no significant differences between two waves (Table S1).

Due to the study inclusion criteria (patients requiring hospitalization due to severe COVID-19) almost all patients required oxygen therapy (96.94% of all patients, comprising 97.33% and 96.75% of the patients from the second and third waves, respectively). Fifty-five patients (24.02%) required high flow nasal oxygen therapy (HFNO), comprising 20 (26.67%) and 35 (22.73%) patients in the second and third waves, respectively. Mechanical ventilation (MV) was necessary in 9.61% of patients, comprising 14.67% and 7.14% in the second and third waves, respectively. There were no significant differences regarding the need for conventional oxygen therapy, HFNO, MV, the overall length of oxygen therapy, maximum oxygen flow in conventional therapy, and HFNO and FiO2 in HFNO between the two analyzed waves.

Thirty-one patients (13.54%) required ICU admission, comprising 15 patients (20%) from the second wave and 16 patients (10.39%) from the third wave, with no statistically significant difference between two waves (*p* = 0.074). The median period from the onset of symptoms to ICU admission was 10 days in both groups. There was also no significant difference regarding the need for vasopressors and CRRT. Sixteen patients (6.99%) died, comprising eight (10.67%) patients from the second wave and eight (5.19%) patients from the third wave, with no statistically significant difference between the two waves (*p* = 0.212). There was no statistically significant difference in ICU mortality between the groups (*p* = 0.862), with rates of 53.33% and 43.75% for the second and third waves, respectively (Table 1, Table 2 and Table S1).

Leukocyte (white blood cell, WBC), neutrophil and platelet (PLT) counts, neutrophil and immature granulocyte (IG) percentages, the frequency of leukocytosis (defined as WBC count > 10 × 10^3^/μL) and neutrophilia (defined as neutrophil count > 7 × 10^3^/μL), the neutrophil-to-lymphocyte ratio (NLR), prothrombin time (PT) and INR at admission were higher; and the lymphocyte percentages and activated partial thromboplastin time (APTT) at admission were lower in the second wave compared with the third wave, while these differences were not statistically significant at the 7th DOH. C-reactive protein (CRP) at the 7th DOH was significantly higher in the second wave compared with the third wave. However, this difference was not statistically significant after the removal of the outliers, and there was no significant difference between the two waves regarding CRP levels at admission. Procalcitonin (PCT) was significantly higher, while albumin and myoglobin were significantly lower in the second wave compared with the third wave at the 7th DOH, but not at admission. There were no statistically significant differences between the two waves regarding interleukin-6 (IL-6), ferritin, antithrombin III (AT III), D-Dimer at admission, fibrinogen, lactate dehydrogenase (LDH), calcium and vitamin D3 levels, nor in the levels of cardiac, renal, and liver injury markers. However, D-Dimer at the 7th DOH was significantly higher, and D-Dimer > 500 µg/L FEU at the 7th DOH was significantly more frequent in the second wave than in the third wave. There were no differences in red blood cell (RBC) and lymphocyte counts, the frequency of lymphopenia, thrombocytopenia (defined as PLT count below 150 × 10^3^/μL), and thrombophilia (defined as PLT count above 400 × 10^3^/μL), and hemoglobin and hematocrit levels (Table 3, Table 4, Tables S2 and S3).

### 3.2. Factors Associated with Poor Prognosis

**Figure 1:**
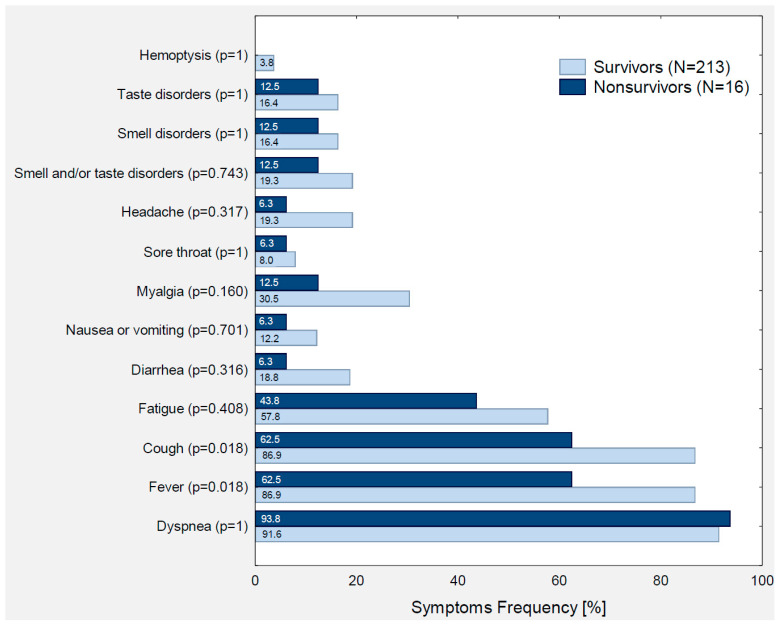
Comparison of symptoms frequency between survivors and non-survivors (presented as percentages, with *p*-values for each comparison).

**Figure 2:**
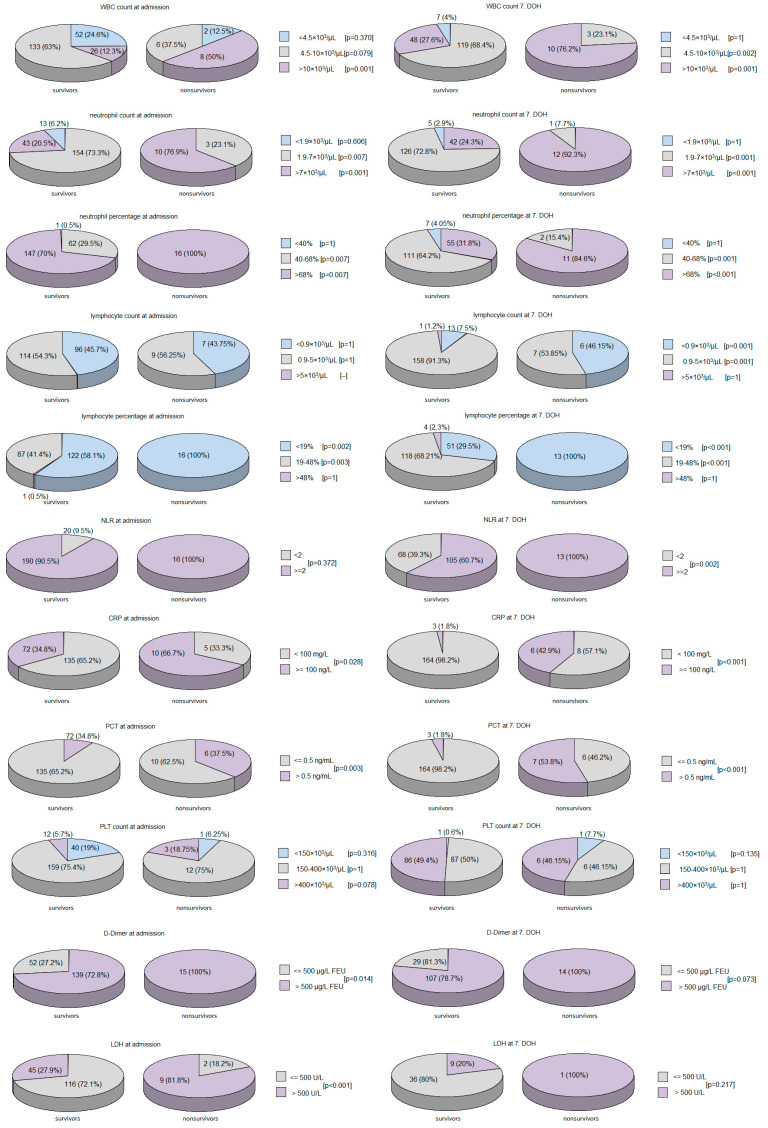
Comparison of patients’ laboratory parameters (as categorical variables) between survivors and non-survivors. Variables are presented as the number of patients and percentages and compared with the chi-squared test or Fisher’s exact test as appropriate. *p*-values are presented for each comparison. A two-sided *p*-value < 0.05 was considered statistically significant. Leukocytosis and NLR ≥ 2 at 7. DOH, and neutrophilia, lymphopenia, CRP ≥ 100 mg/L and PCT > 0.5 ng/mL at admission and at 7. DOH were found more frequently in non-survivors compared to survivors, indicating a hyperinflammatory reaction. Moreover, a higher prevalence of D-Dimer levels greater than 500 µg/L FEU at admission may indicate hypercoagulability, while a higher prevalence of LDH levels above 500 U/L at admission may reflect more pronounced tissue damage in non-survivors.

A comparison of the general, clinical, and laboratory characteristics between the survivors and non-survivors is presented in Table 4, Table 5, Table 6 and Table S4 and Figure 1 and Figure 2.

There were no significant age (*p* = 0.152) and sex (*p* = 0.236) differences between the survivors and non-survivors. There were also no differences between these groups regarding the frequencies of any of the blood types. Weight and BMI were higher in the non-survivors compared to survivors. However, these differences were not significant after the removal of the outliers.

Comorbidities were more frequent in the non-survivors compared to survivors (68.75% vs. 38.5%, *p* = 0.035). Moreover, chronic arrhythmia was significantly more frequent in the non-survivors than in survivors, while there were no statistically significant differences in the frequency of insulin resistance, diabetes, hypertension, asthma, hypothyroidism, or Hashimoto disease. A history of current or former smoking was significantly more frequent among non-survivors than survivors, while there was no significant difference between these groups regarding current smoking status.

Regarding the reported symptoms, fever and cough were significantly more frequent among the survivors than non-survivors. However, this may be due to the poorer availability of data on baseline symptoms in patients who were at the critical stage of COVID-19 at admission and should be interpreted with caution. There were no significant differences regarding other initial symptoms, such as dyspnea, fatigue, diarrhea, nausea and vomiting, myalgia, sore throat, headache, smell and/or taste disorders, and hemoptysis.

The percentage of lung involvement on CT was significantly higher and lung involvement of at least 50% was more frequent in the non-survivors than in survivors.

SpO_2_ at admission was significantly lower and the maximum oxygen flow needed in conventional oxygen therapy was significantly higher in the non-survivors than in survivors. Non-survivors required HFNO, MV, ICU admission, vasopressors, and CRRT more frequently than survivors.

WBC count, neutrophil count, neutrophil percentage, NLR, IG count, and IG percentage were significantly higher, leukocytosis and neutrophilia were more frequent, and the lymphocyte percentage was lower in the non-survivors than in survivors, both at admission and at the 7th DOH. The lymphocyte count was significantly lower, and lymphopenia (defined as a lymphocyte count below 0.9 × 10^3^/μL) and NLR of at least 2 were significantly more frequent in the non-survivors than in survivors at the 7th DOH, but not at admission.

CRP, PCT, and IL-6 levels were significantly higher, and CRP > 100 mg/L and PCT > 0.5 ng/mL were significantly more frequent in non-survivors compared to survivors at admission and at the 7th DOH. There were no significant differences between non-survivors and survivors regarding ferritin and AT III levels.

D-Dimer at admission and at the 7th DOH was significantly higher and D-Dimer > 500 µg/L FEU at admission was significantly more frequent in non-survivors compared to survivors. APTT at admission, but not at the 7th DOH, was significantly longer in survivors compared to non-survivors, while there were no significant differences between these groups regarding the PLT count, PT, and fibrinogen. LDH levels at admission were significantly higher and LDH > 500 U/L at admission was significantly more frequent in non-survivors than in survivors.

There were no significant differences regarding RBC counts and hematocrit and hemoglobin levels at admission. However, at the 7th DOH these parameters were significantly lower in the non-survivors than in survivors.

High-sensitive troponin I (hs-TnI) and N-terminal-pro-B-type natriuretic peptide (NT-proBNP) levels were significantly higher in non-survivors than in survivors at admission and at the 7th DOH. The creatine kinase-myocardial band (CK-MB) and myoglobin levels were significantly higher in non-survivors than in survivors at admission, while at the 7th DOH these differences were not significant. The creatine kinase (CK) levels at the 7th DOH were significantly higher in non-survivors than in survivors, while this difference was not significant at admission.

Urea levels were significantly higher in non-survivors than in survivors at admission and at the 7th DOH, while creatinine levels were significantly higher, and the estimated glomerular filtration rate (EGFR) was significantly lower in non-survivors than in survivors at admission, but not at the 7th DOH.

Alanine aminotransferase (ALT) levels at the 7th DOH, but not at admission, were significantly higher in survivors compared with non-survivors. However, there were no other statistically significant differences between these groups regarding liver injury markers, such as aspartate aminotransferase (AST), gamma-glutamyl transferase (GGT), and/or total bilirubin levels.

Albumin concentrations were significantly lower in non-survivors than in survivors at the 7th DOH, while this difference was not significant at admission.

Total calcium concentrations were significantly lower in non-survivors than in survivors at admission and at the 7th DOH, while vitamin D3 levels were significantly lower in non-survivors than in survivors at admission, but not at the 7th DOH.

#### 3.2.1. Correlations between Patients’ Characteristics and the Need for MV and ICU Treatment

Correlations between patients’ characteristics and the need for MV and ICU treatment are presented in Tables S5–S7.

There was a weak association between weight, BMI, BMI above 40 kg/m^2^, and comorbidities and the need for MV. There were also weak associations between diabetes and smoking and the need for MV and ICU treatment. There was a positive correlation between the percentage of lung involvement on CT and the maximum oxygen flow in conventional oxygen therapy and a negative correlation between SpO_2_ at admission, and the need for MV and ICU treatment. The need for HFNO, ICU treatment, vasopressors, and CRRT were associated with the need for MV, and the need for HFNO, MV, vasopressors, and CRRT were associated with the need for ICU treatment.

WBC, neutrophil and IG counts, neutrophil percentage, NLR, CRP, PCT, and IL-6 levels were positively correlated with the need for MV and ICU treatment, and there was an association between leukocytosis and neutrophilia, CRP > 100 mg/L and PCT > 0.5 ng/mL (at admission and at the 7th DOH) and the need for MV and ICU treatment. There was also a weak negative correlation between the lymphocyte count at the 7th DOH and the need for MV and ICU treatment, and an association of lymphopenia at the 7th DOH and the need for MV and ICU treatment. Ferritin levels at admission were positively correlated with the need for MV, while ferritin levels at the 7th DOH—with the need for ICU treatment. RBC counts, hematocrit and hemoglobin levels at the 7th DOH were negatively correlated with the need for MV and ICU treatment.

PLT count and thrombophilia at admission were positively correlated with the need for MV and ICU treatment, and thrombocytopenia at admission with the need for ICU treatment. There was a positive correlation between D-Dimer, and an association of D-Dimer > 500 µg/L FEU (at admission and at the 7th DOH), and the need for MV and ICU treatment.

There was a positive correlation between LDH levels (at admission and at the 7th DOH), and an association of LDH > 500 U/L at admission and the need for MV and ICU treatment. The need for MV and ICU treatment were also correlated positively with the hsTnI and NT-pro-BNP levels at admission and at the 7th DOH, CK-MB and myoglobin levels at admission, and CK levels at the 7th DOH, and there was a positive correlation between CK levels at admission and the need for ICU treatment, but not MV. Urea levels were correlated positively with the need for MV and ICU treatment, and creatinine levels at admission with the need for MV. There was a moderate to strong negative correlation between albumin and calcium concentrations (at admission and at the 7th DOH) and the need for MV and ICU treatment, and between vitamin D3 levels at the 7th DOH and the need for ICU treatment.

#### 3.2.2. Predictors of Death in Logistic Regression

Univariate logistic regression revealed that weight > 100 kg, BMI ≥ 40 kg/m^2^, comorbidities, diabetes or insulin resistance, chronic arrhythmia, CK-MB > 20 U/L at admission and at the 7th DOH, CK > 190 U/L at the 7th DOH, D-Dimer > 500 µg/L FEU at admission and at the 7th DOH, EGFR < 60 mL/min at admission and at the 7th DOH, GGT > 120 U/L at admission and at the 7th DOH, hematocrit < 40% at the 7th DOH, hemoglobin < 12 g/dL at the 7th DOH, creatinine > 1.2 mg/dL at admission and at the 7th DOH, LDH > 500 U/L at admission, urea > 49 mg/dL at the 7th DOH, NT-proBNP > 190 pg/mL at admission and at the 7th DOH, RBC count < 4.5 × 10^6^/μL at the 7th DOH, hsTnI > 34 pg/mL at the 7th DOH, total calcium < 2.1 mmol/L at admission and at the 7th DOH, NLR ≥ 2 at the 7th DOH, lymphocyte count < 0.9 × 10^3^/μL at the 7th DOH, lymphocyte percentage < 19% at the 7th DOH, neutrophil count > 7 × 10^3^/μL at admission and at the 7th DOH, neutrophil percentage > 68% at the 7th DOH, WBC count > 10 × 10^3^/μL at admission and at the 7th DOH, CRP ≥ 100 mg/L at admission and at the 7th DOH > 100, and PCT > 0.5 ng/mL at admission and at the 7th DOH, were associated with increased risk of death (Table 7 and Table 8). Comorbidities, WBC count > 10 ×10^3^/μL, and PCT > 0.5 ng/mL were associated with death in multivariate analysis for variables obtained at admission (Table 8).

#### 3.2.3. Predictors of MV in Logistic Regression

Univariate logistic regression revealed that weight > 100 kg, BMI ≥ 40 kg/m^2^, comorbidities, diabetes or insulin resistance, chronic arrhythmia, CK-MB > 20 U/L at admission and at the 7th DOH, CK > 190 U/L at admission and at the 7th DOH, D-Dimer > 500 µg/L FEU at admission and at the 7th DOH, EGFR < 60 mL/min at admission and at the 7th DOH, GGT > 120 U/L at admission and at the 7th DOH, hematocrit < 40% at the 7th DOH, hemoglobin < 12 g/dL at the 7th DOH, creatinine > 1.2 mg/dL at admission and at the 7th DOH, LDH > 500 U/L at admission, urea > 49 mg/dL at admission and at the 7th DOH, NT-proBNP > 190 pg/mL at admission and at the 7th DOH, RBC count < 4.5 × 10^6^/μL at the 7th DOH, hsTnI > 34 pg/mL at admission and at the 7th DOH, total calcium < 2.1 mmol/L at admission and at the 7th DOH, NLR ≥ 2 at the 7th DOH, lymphocyte count < 0.9 × 10^3^/μL at the 7th DOH, lymphocyte percentage < 19% at the 7th DOH, neutrophil count > 7 × 10^3^/μL at admission and at the 7th DOH, neutrophil percentage > 68% at the 7th DOH, WBC count > 10 × 10^3^/μL at admission and at the 7th DOH, CRP > 100 mg/L at admission and at the 7th DOH, and PCT > 0.5 ng/mL at admission and at the 7th DOH, were associated with increased risk of MV (Table 7 and Table 9). BMI ≥ 40 kg/m^2^, LDH > 500 U/L, WBC count > 10 × 10^3^/μL, and PCT > 0.5 ng/mL, were significantly associated with MV in multivariate analysis for variables obtained at admission (Table 9).

#### 3.2.4. Predictors of ICU Treatment in Logistic Regression

Univariate logistic regression revealed that weight > 100 kg, BMI ≥ 40 kg/m^2^, diabetes or insulin resistance, CK-MB > 20 U/L at admission and at the 7th DOH, CK > 190 U/L at admission and at the 7th DOH, D-Dimer > 500 µg/L FEU at admission and at the 7th DOH, EGFR < 60 mL/min at admission and at the 7th DOH, GGT > 120 U/L at the 7th DOH, hematocrit < 40% at the 7th DOH, hemoglobin < 12 g/dL at the 7th DOH, creatinine > 1.2 mg/dL at admission and at the 7th DOH, LDH > 500 U/L at admission, urea > 49 mg/dL at the 7th DOH, NT-proBNP > 190 pg/mL at admission and at the 7th DOH, RBC count < 4.5 × 10^6^/μL at the 7th DOH, hsTnI > 34 pg/mL at admission and at the 7th DOH, total calcium < 2.1 mmol/L at admission and at the 7th DOH, NLR ≥ 2 at the 7th DOH, lymphocyte count < 0.9 × 10^3^/μL at the 7th DOH, lymphocyte percentage < 19% at admission and at the 7th DOH, neutrophil count > 7 × 10^3^/μL at admission and at the 7th DOH, neutrophil percentage > 68% at admission and the 7th DOH, WBC count > 10 × 10^3^/μL at admission and at the 7th DOH, CRP > 100 mg/L at admission and at the 7th DOH, and PCT > 0.5 ng/mL at admission and at the 7th DOH were associated with increased risk of ICU treatment (Table 7 and Table 10). D-Dimer > 500 µg/L FEU, LDH > 500 U/L, WBC count > 10 × 10^3^/μL, and PCT > 0.5 ng/mL were significantly associated with ICU treatment in multivariate analysis for variables obtained at admission (Table 10).

#### 3.2.5. ROC Analysis

**Figure 3:**
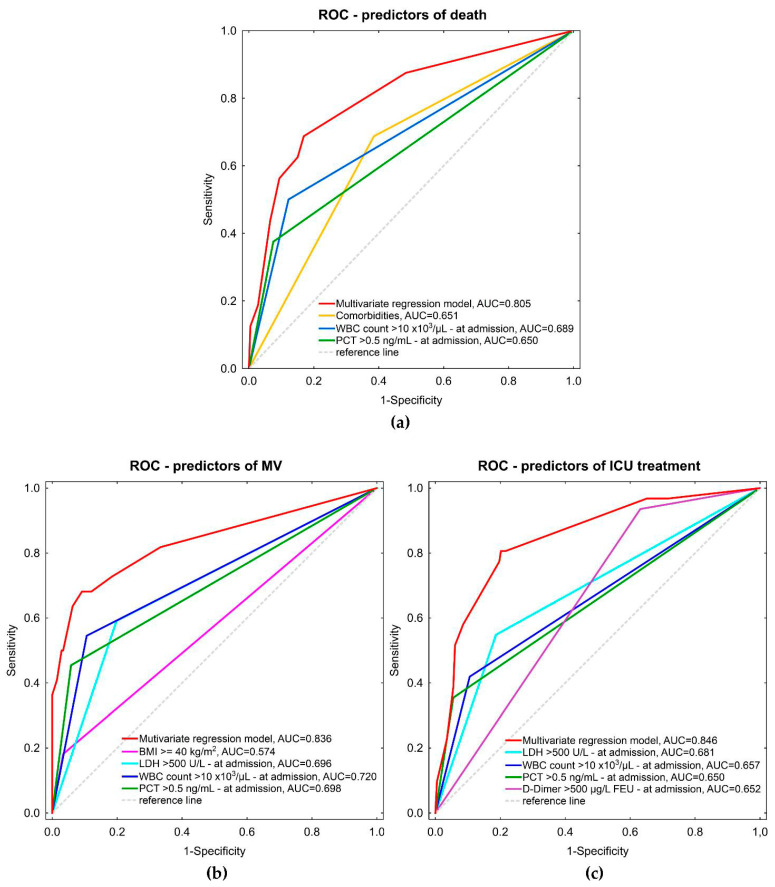
The receiver operating characteristic (ROC) curves with an area under the curve (AUCs) of individual factors and combined models in multivariate logistic regression analysis for predicting: (**a**) death; (**b**) MV; and (**c**) ICU treatment. Nominal data (including comorbidities) and categorized clinical and laboratory parameters obtained at admission and significant in univariate regression were included in this analysis. Comorbidities (AUC = 0.651), WBC count > 10 × 10^3^/μL (AUC = 0.651), and PCT > 0.5 ng/mL (AUC = 0.650), were associated with death; BMI ≥ 40 kg/m^2^ (AUC = 0.574), LDH > 500 U/L (AUC = 0.696), WBC count > 10 × 10^3^/μL (AUC = 0.720), and PCT > 0.5 ng/mL (AUC = 0.698) were associated with MV, while D-Dimer > 500 µg/L FEU (AUC = 0.652), LDH > 500 U/L (AUC = 0.681), WBC count > 10 × 10^3^/μL (AUC = 0.657), and PCT > 0.5 ng/mL (AUC = 0.650), were associated with ICU treatment. The combined multivariate regression models for predicting death, MV and ICU treatment had the AUC values of 0.805, 0.836, and 0.846, respectively.

The combined multivariate regression models for predicting death, MV, and ICU treatment had area under the curve (AUCs) values of 0.805, 0.836, and 0.846, respectively. The ROC curves with AUCs of combined models and individual factors are presented in Figure 3.

## 4. Discussion

The SARS-CoV-2 alpha variant has been shown to be more transmissible than the wild-type variants [11,12]. However, its impact on disease severity remains unclear [13,14,15,16,18,39].

Challen et al., in a cohort study of 54,906 matched pairs [15], found that the hazard ratio of death within 28 days associated with infection with the alpha variant, compared with the wild-type variants, was 1.64. However, this study only included patients older than 30 years. Moreover, Davies et al. [14] analyzed a dataset of positive SARS-CoV-2 community tests, identifying 4945 deceased patients with known SGTF status, and estimated the hazard of death associated with the alpha variant to be 61% (42–82%) higher than for those with pre-existing variants. Furthermore, Grint et al. [40] found an increased risk of death in SGTF (S gene target failure, indicating the B.1.1.7 variant) compared to non-SGTF cases. However, the age range in the youngest group in an age subgroup analysis was as wide as 0–59 years. Moreover, although an initial analysis of CO-CIN data reported by NERVTAG [39] did not identify increased in-hospital case-fatality rate associated with the alpha variant, several other unpublished analyses summarized in NERVTAG were consistent in reporting increased disease severity in people infected with the alpha variant compared to variants then considered as non-VOCs [39].

On the contrary, a study by Frampton et al. [13] found no association of severe disease and death with B.1.1.7 compared to the non-B.1.1.7 lineage in hospitalized COVID-19 patients. Similarly, Brookman et al. [18] found no evidence of a greater disease severity in children hospitalized during the second wave in England (1 March to 31 May 2020) compared to the first wave (1 November 2020, to 19 January 2021), which prompted them to suggest that there is no appreciably different clinical course of the infection with the B.1.1.7 variant compared to the original variant. It should be noted that these studies showing no increased severity and mortality associated with the alpha variant only included hospitalized patients [13,18,39]. It is therefore also possible that the SARS-CoV-2 alpha variant could have been associated with more severe disease compared to previous variants, increasing the number of patients severe enough to meet hospital admission criteria, but had no impact on the in-hospital outcomes, including mortality [39]. Indeed, Nyberg et al. found that the risk of hospitalization was higher for people infected with the alpha variant compared with wild-type variants, with an adjusted hazard ratio of hospital admission of 1.52 [17]. Moreover, although Martin-Blondel et al. reported a greater severity of the disease associated with the alpha variant in hospitalized patients, this result was statistically significant when defining severe disease as a WHO-scale  >  5 or the need of a non-rebreather mask, while the differences in the mortality rate and the need for HFNO, ICU admission and MV or ECMO were not statistically significant [16]. It is also noteworthy that the alpha variant has been de-escalated from being VOC, indicating that it no longer poses a significant risk to public health [7].

In our study, we found no significant differences in disease severity or mortality between the second and third wave patients, which may indicate that in hospitalized severely ill young adults, the SARS-CoV-2 alpha variant did not increase the incidence of critical disease or death. Indeed, we found no significant differences between the two waves regarding mortality and the need for ICU. There were also no differences between the second and third waves regarding the median percentage of lung involvement on CT at admission and the need for MV. Therefore, our results indicate that the alpha variant does not appear to be associated with worse outcomes in hospitalized young adults than the wild-type variants.

It is noteworthy that we have found no significant differences between the second and third waves regarding sex, BMI, smoking status, and comorbidities. Moreover, in our study WBC, neutrophil and IG counts, IG percentages, NLR and the frequency of leukocytosis and neutrophilia at admission were higher, and the lymphocyte percentage at admission was lower in the second wave compared with the third wave, while there were no significant differences regarding IL-6, CRP, and ferritin levels between these two waves. Furthermore, although there was no significant difference between waves in terms of PCT levels at admission, PCT levels at the 7th DOH were significantly higher in the second wave compared with the third wave. As these markers of hyperinflammation seem to be associated with a worse outcome, as discussed below, these findings further contradict a greater disease severity due to the alpha SARS-CoV-2 variant compared to the wild-type variants. Moreover, high D-Dimer and low albumin levels also appear to be associated with poor prognosis, and we found no significant differences between waves in terms of D-Dimer and albumin levels at admission, while at the 7th DOH D-Dimer levels were even higher and albumin levels lower in the second wave compared with the third wave. In addition, although myoglobin levels at the 7th DOH were significantly lower in the second wave compared with the third wave, there were no differences between waves in other, more cardiac-specific biomarkers. Hence, this finding is not likely to indicate more pronounced myocardial injury in the third wave.

Regarding the delta variant, although it is generally considered to be associated with greater disease severity, data are inconsistent [20,21,22,23]. Two studies in the UK showed a greater risk of hospitalization with the delta variant compared to the alpha variant. However, these studies did not report on disease severity or mortality [41,42]. A study from Canada compared then-considered VOC and non-VOC strains, and found increased risk of hospitalization, ICU admission, and death with N501Y-positive variants (alpha, beta, and gamma variants) and even more pronounced for the delta variant [20]. On the other hand, a US study comparing children suffering from COVID-19 during the delta vs. pre-delta eras found no significant difference in hospitalization rates and lower odds of severe disease [22]. Moreover, Kläser et al. [23] found that illness duration was lower in those infected with delta compared to alpha variant, though unchanged in unvaccinated patients, and there was no difference regarding hospitalization.

Omicron variant, currently labeled as VOC, appears to cause less severe disease [8,43], however, more data are still needed [27]. In a study by Lauring et al. [43] the severity was higher for delta than alpha, and lower for omicron than delta variants. Sievers et al. [25] found significantly reduced odds of hospitalization, ICU admission and death in patients infected with omicron compared to delta variant, whereas Van Goethem et al. [26] reported significantly lower risk for severe COVID-19 and ICU admission in hospitalized patients infected with the omicron compared to delta variant, while in-hospital mortality was not significantly different. Wolter et al. [27] found reduced risk of severe disease among patients infected with SGTF (as a proxy for the omicron variant) compared with individuals with earlier delta variant infections, however, the authors did not find the difference in severity between SGTF and non-SGTF infections diagnosed during the same time period, and suggested that immunity, due to previous infection, vaccination, or both, may at least in part account for the reduced severity of omicron compared to delta variant infections. Indeed, continuously changing vaccination status makes these comparisons of SARS-CoV-2 variants even harder. Furthermore, even in non-vaccinated patients the severity of the disease may now be more and more frequently affected by other factors, including prior SARS-CoV-2 infection. Nevertheless, it seems possible that the omicron will share the fate of the previous variants, and be de-escalated.

SARS-CoV-2 infection may be asymptomatic or symptomatic, with the course of the disease varying widely from mild to severe to critical. A report by the Chinese Center for Disease Control and Prevention in the initial period of the pandemic (up to 11 February 2020), based on 44,500 confirmed COVID-19 cases, showed that mild disease was found in 81% of cases, severe disease in 14%, and critical disease (with respiratory failure, septic shock and/or multiple organ failure) in 5%, with an overall case-fatality rate of 2.3% and no deaths among noncritical patients [30]. Similarly, in a CDC report analyzing cases reported between 22 January and 30 May 2020, 14% of patients required hospitalization, 2% were admitted to the ICU, and 5% died [44]. It is noteworthy that initially non-severe COVID-19 patients may progress in approximately a week. In our study, the median time from the onset of symptoms to hospital admission was eight days in the second wave and nine days in the third wave, and the median time from the onset of symptoms to ICU admission was 10 days in both groups. Similarly, in a systematic review by Xie et al. [45], the median time from the onset of the disease to first hospital admission was seven days, dyspnea occurred after 5–8 days, and ARDS after 8–9 days, and the median time to ICU admission was 10.5 days. In young adults, Liu et al. reported a median time from the onset of symptoms to hospital admission of 11 days and 10 days in the survivors and non-survivors, respectively [31], while in a study by Owusu et al. this time period was seven days [46].

Regarding inpatients, in a study of 16,000 adults hospitalized with COVID-19 from March to December 2020, the percentage of patients admitted to the ICU decreased from 37.8% in March to 20.5% in December, and the overall fatality rate was 11.4% [47]. In another study [48], out of 2634 patients hospitalized for COVID-19 between 1 March and 4 April 2020, 14.2% were admitted to the ICU and 21% died.

In the general population, certain demographic and clinical features have been associated with the risk for severe course of COVID-19 including older age [28,29,48,49,50,51,52,53,54], male sex [48,52,53], smoking [53,54,55], obesity [51,52,53,55], and other comorbidities [44,55] such as diabetes [44,51,54,55], hypertension [30,51], heart conditions [30,52,55], chronic respiratory diseases [30,55], chronic kidney disease [52,55], and cancer [20,53,55]. As already mentioned, data on clinical features and risk factors of severe COVID-19 in young adults are scarce.

Current evidence indicates that older adults are at risk of having more severe disease. In a study by Verity et al. [49] the hospitalization rate was 1.04% in patients aged 20–29, 3.43% in patients aged 30–39, 4.25% in patients aged 40–49, 8.16% in patients aged 50–59, 11.8% in patients aged 60–69, 16.6% in patients aged 70–79, and 18.4% in patients aged 80 or over. Luo et al., after dividing their study population into four age groups, found severe and critical disease, respectively, in none of the children (18 years or younger), 1.5% and 0.8% of young adults (19–44 years), 6.5% and 6.5% of middle-aged adults (45–64 years), and 12.7% and 20.3% of elderly adults (65 years or older) [50]. Age also appears to be associated with increased mortality [28,29,48,49,50]. In the aforementioned study by Verity et al. [49], the adjusted case-fatality ratio was estimated at 0.06% in patients aged 20–29, 0.15% in patients aged 30–39, 0.3% in patients aged 40–49, 1.25% in patients aged 50–59, 3.99% in patients aged 60–69, 8.61% in patients aged 70–79, and 13.4% in patients aged 80 or over. Williamson et al. [28] found a greater than 20-fold-increased risk of death in patients 80 years and older compared to 50–59-year-olds. For these reasons, many studies of COVID-19 focus either on general epidemiological data or on older populations [29,33,56]. However, COVID-19 may also result in severe disease and death in young adults [33,57,58,59]. In our study, 13.54% of patients required ICU admission and 7% died, which agrees with other studies of young adults. Indeed, in a study of 395 patients aged 18–35 years hospitalized for COVID-19, 21% required invasive mechanical ventilation and 13.9% died [33]. In a study of SARS-CoV-2-positive 18–45-year-olds, who had presented to emergency departments, 9% died during hospitalization [57]. Richardson et al. [58] found that the 30-day in-hospital mortality in COVID-19 patients aged 18–39 years was 4.9%, while Cunningham et al. [59] found that of the 3222 18–34-year-olds hospitalized for COVID-19, 21% were admitted to the ICU and mortality was 2.7%. Therefore, it is essential to identify the clinical features and risk factors for severe COVID-19 in this age group. Of note, we have not found a significant age difference between survivors and non-survivors, possibly because our study only involved young adults. Similarly, in a study by Cunningham et al. [59], the odds of MV or death did not vary significantly with age. We believe that studies of young adults are of great importance due to the small influence of other factors, such as comorbidities, on the course of infection in this age group, which may be useful in determining the influence of the causative variant on disease severity.

The male sex of young adults has been related with poor prognosis by some authors [31,35,59], while others have failed to find this relationship [58,60]. In our study, there was no significant difference between the survivors and non-survivors regarding sex. However, it should be noted that we observed a male predominance in both waves, which may indicate that men were more likely to have a disease severe enough to require hospital admission.

Some studies in young adults found no association of smoking and mortality [33,58,61]. Conversely, in our study, a history of current or former smoking, but not current smoking, was significantly more frequent among the non-survivors than survivors, and we have found a weak positive correlation between smoking and the need for MV and ICU treatment. Moreover, according to the CDC, there is evidence that in the general population smoking is associated with a higher risk of severe COVID-19 [55]. Therefore, in our opinion, smoking history should be taken into consideration while assessing the possible risk factors for severe COVID-19 in young adults.

Higher BMI and obesity have been identified as risk factors for poor prognosis in young adults in several studies [33,35,57,58,59,61]. Although we have also found weight and BMI to be higher in non-survivors compared to survivors, these differences were not significant after the removal of the outliers. However, it should be noted that in our study of COVID-19 patients, all of whom were hospitalized due to severe disease, the median BMI was 30.58 kg/m^2^ (IQR 27.1–34.3 kg/m^2^), and normal BMI (below 25 kg/m^2^) was found in only 12.34%. Moreover, we have also found a positive correlation between BMI and the need for MV. Furthermore, weight > 100 kg and BMI ≥ 40 kg/m^2^ were significant predictors of death, MV and ICU treatment in univariate logistic regression, and BMI ≥ 40 kg/m^2^ was also a significant predictor of MV in multivariate analysis. Therefore, it appears that overweight or obesity should also be considered risk factors for poor prognosis, possibly due to their association with other comorbidities, such as reduced lung volumes and hypercoagulable states [58].

In our study, comorbidities were significantly more frequent in non-survivors compared to survivors, and were associated with the risk of death and MV in univariate logistic regression, and with the risk of death in multivariate analysis. Similarly, Richardson et al. [58] found a Charlson comorbidity index score to be an independent predictor of in-hospital 30-day mortality in young adults hospitalized for COVID-19. We have also found that chronic arrhythmia was significantly more frequent in the non-survivors than in the survivors, while for insulin resistance, diabetes, and hypertension, although their frequency was greater in non-survivors than in survivors, these differences were not significant (*p* = 0.058, 0.199 and 0.268, respectively). There were no significant differences in the frequency of asthma, hypothyroidism, and Hashimoto disease (*p* = 1, 0.485 and 1, respectively). In univariate logistic regression, having diabetes or insulin resistance was significantly associated with the risk of death, MV, and ICU treatment, and chronic arrhythmia was significantly associated with the risk of death and MV. Several studies of young adults have identified diabetes as a risk factor for more severe disease [31,33,35,59,60,61]. Hypertension has been associated with poor prognosis in some studies [31,33,59,61], however, others have not found this association [35,58,60]. Similarly, asthma was predictive of more severe disease in some studies [35,60], but other authors found it was not associated with increased mortality [33,58]. Additionally, cardiac [33,35] and renal [33,35] conditions have been identified as related to poor prognosis in some studies, while no association of thyroid diseases and severe COVID-19 has been found [35,60]. Overall, the association of comorbidities and the outcome of SARS-CoV-2 infection appears to be less pronounced in young adults than in the general population, as a greater number of comorbidities in elderly patients may lead to a more complex pathogenesis in COVID-19 and its complications [34].

In our study, the percentages of lung involvement and lung involvement of at least 50% on CT were significantly higher in the non-survivors than in survivors. There was also a weak positive correlation between the percentage of lung involvement and the need for MV and ICU treatment. This agreed with a study by Ruch et al. [62] which found that the extent of changes on initial CT was associated with prognosis, with 69.5% of patients who had lung involvement over 50% having developed severe disease, compared to 22.9% of patients with lung involvement no greater than 25%. Similarly, in a study by Annoni et al. [63] the percentage of damaged lung parenchyma volume on CT was correlated with the course of COVID-19, with average infected lung volume significantly higher in the non-survivors.

In our study, SpO_2_ at admission was significantly lower in non-survivors than in survivors, and there was a weak to moderate negative correlation between SpO_2_ at admission and the need for MV and ICU treatment. Moreover, we found a more frequent need for HFNO and MV and a higher maximum oxygen flow, in both conventional oxygen therapy and HFNO, in non-survivors than in survivors. This agreed with previous studies among young adults reporting an association of respiratory distress and mortality [31,33,34]. Furthermore, as predicted, we found that ICU admission, vasopressors, and CRRT were more frequent in non-survivors than in survivors.

Current evidence in the general population indicates that a number of laboratory anomalies may be associated with the risk for severe course of COVID-19 and worse outcomes, including elevated WBC [29,54,64,65,66,67,68,69] and neutrophil counts [64,65,66,68,69,70], lymphopenia [29,52,61,64,65,66,68,69,70], elevated NLR [64,66], thrombocytopenia [29,64,68,69,70,71], increased inflammatory markers, including CRP [52,64,65,66,68,69,70,71], PCT [29,52,66,68,69,70,71], and ferritin [29,64,65,66,68,69,70], and inflammatory cytokines, such as IL-6 [29,64,65,68,69,70], as well as organ and coagulation dysfunction markers, including elevated LDH [29,64,65,66,68,69,70,71], troponin and hs-TnI [29,31,52,68,69], NT-proBNP [66,68,70], creatinine [29,52,68,69,70,71], CK [29,68,71], liver enzymes [29,65,66,68,69,70,71], D-Dimer [29,52,64,65,66,67,68,69,70,71], longer prothrombin time [29,64,65,68,69], and decreased serum albumin levels [29,65,66,68,69,70]. However, Luo et al. [50] found many laboratory parameters to be significantly different in younger compared to older COVID-19 patients, including higher WBC, lymphocyte and PLT counts, and hemoglobin and albumin levels, and lower levels of CRP, ALT, creatinine, and D-dimer. Similarly, Liu et al. [31] found many significantly different laboratory parameters in younger (defined as younger than 60 years old) compared to older COVID-19 patients, including higher lymphocyte counts and albumin levels, and lower neutrophil counts, NLR, PT, and levels of CRP, PCT, D-dimer, LDH, creatinine, and NT-proBNP. These differences are thought to result from higher incidences of organ dysfunctions and comorbidities, as well as poorer immune responses in older individuals [31,50]. Therefore, the predictors of severe disease and mortality in younger COVID-19 patients seem to differ from those in general population.

SARS-CoV-2 infection causes a host immune response, which in most patients will contribute to viral elimination. However, in some cases, the activation of nucleic acid sensors on lung epithelium and alveolar macrophages triggers the elevated release of cytokine and other proinflammatory mediators, such as IL-1, IL-6, and tumor necrosis factor-α (TNF-α), resulting in the recruitment and infiltration of neutrophils, monocytes, and other leukocytes. Moreover, these cytokines stimulate bone marrow to produce and release immature granulocytes that infiltrate the lungs, further increasing the exuberant inflammatory reaction [72,73]. In addition, recruited neutrophils kill pathogens by producing reactive oxygen species (ROS) and releasing web-like structures consisting of DNA and antimicrobial agents, such as and myeloperoxidase and histones, known as the neutrophil extracellular traps (NETs). However, this may also cause destruction of infected tissue, microthrombosis, and organ damage [73,74]. Furthermore, the release of cytokines, including IL-6, induces the synthesis of acute phase proteins, such as CRP, fibrinogen, and ferritin [72], which, in turn, may further affect the immune reaction, being able to induce the expression of both pro- and anti-inflammatory mediators [75,76]. Moreover, the exacerbated inflammatory reaction may lead to cytokine-induced lymphocyte apoptosis [74,77,78]. Other possible causes of lymphopenia in COVID-19 include a direct viral infection of ACE2-expressing lymphocytes, destruction of lymphatic organs, and increased lymphocyte consumption in the infected tissues [74,77,78]. For these reasons, an elevated neutrophil to leukocyte ratio (NLR) is also observed in COVID-19 [74]. Indeed, in our study, we found that the WBC, neutrophil, and IG counts, IG percentages, the incidence of leukocytosis, neutrophilia, and lymphopenia, NLR, and IL-6 levels were significantly higher in non-survivors than in survivors. There was also a positive correlation between the WBC, neutrophil an IG count, neutrophil and IG percentages, leukocytosis, neutrophilia, NLR, and IL-6 levels and the need for MV and ICU treatment, and a negative correlation between the lymphocyte percentage and the need for MV and ICU treatment. In addition, CRP was significantly higher and CRP above 100 mg/L was significantly more frequent in non-survivors than in survivors, and there was also a weak to moderate positive correlation between CRP and the need for MV and ICU treatment. Furthermore, univariate logistic regression revealed that NLR ≥ 2 and lymphopenia at the 7th DOH, and neutrophilia, leukocytosis, and CRP > 100 mg/L at admission and at the 7th DOH were significantly associated with the risk of death, MV and ICU treatment. In multivariate analysis, CRP > 100 mg/l and leukocytosis at admission were significantly associated with death, MV and ICU treatment. Moreover, although we did not find a significant difference between ferritin levels between non-survivors and survivors, there was a weak positive correlation between ferritin levels at admission and the need for MV, and between ferritin levels at the 7th DOH and the need for ICU treatment.

In accordance with our results, some studies in young adults with COVID-19 found elevated WBC [31,34] and neutrophil counts [31,34], NLR [31] and levels of CRP [31,33,34], and ferritin [34], as well as decreased lymphocyte counts [31,34,60] in deceased cases compared to survivor patients, and elevated neutrophil percentages [79] and CRP [61,79] levels and decreased lymphocyte counts [79] in severe vs. mild patients. However, Altonen et al. [33] found no significant differences in ferritin levels and WBC, neutrophil and lymphocyte counts between survivors and non-survivors, Zhou et al. [79] found lower WBC count and no difference in neutrophil count in severe compared to mild patients, and Maldonado-Cabrera et al. [61] did not observe the increase in NLR in the aggravated COVID-19 patients. Nevertheless, we suggest that the hyperinflammatory reaction may be the reason for the severe course of COVID-19 also in young adults. Furthermore, studies have reported an even stronger influence on poor COVID-19 outcomes of parameters, such as neutrophilia [31] and lymphopenia [60], in young adults than occurring in the elderly, which was attributed to a stronger immune response in young adults with a stronger cytokine storm and hyperinflammatory reaction [31,60].

PCT can be induced directly by bacterial endotoxins and lipopolysaccharides or indirectly through the release of pro-inflammatory cytokines, such as IL-1β, TNF-α and IL-6. However, its synthesis may be inhibited by interferon-γ (INF-γ), with increased in viral infection. Therefore, PCT is not typically elevated in mild SARS-CoV-2-infection, while its increase is observed in severe COVID-19, especially due to a bacterial co-infection [80,81,82]. Moreover, PCT upregulates the leukocyte surface markers, cytokines, and reactive oxygen species, further aggravating the inflammatory reaction [80]. This is supported by our study, which found that PCT was significantly higher and PCT above 0.5 ng/mL was significantly more frequent in non-survivors compared to survivors, and that there was a positive correlation between PCT, and the need for MV and ICU treatment.

Univariate logistic regression revealed that PCT > 0.5 ng/mL at admission and at the 7th DOH was associated with increased risk of death, MV and ICU treatment, and PCT > 0.5 ng/mL at admission was also associated with increased risk of death, MV and ICU treatment in multivariate analyses. Similarly, other studies of young adult COVID-19 patients have found higher PCT in non-survivors than in survivors [31,33,34].

Apart from the excessive inflammatory reaction, another possible cause of severe course and poor prognosis in COVID-19 may be thrombosis and coagulopathy [61]. Several factors may contribute to coagulation dysfunction in COVID-19, including the cytokine storm leading to an increased production of platelets and fibrinogen, complement activation, vascular dysfunction, Renin-Angiotensin-Kallikrein-Kinin systems (RAS-KKS) imbalance, and excessive intravascular NETs formation by neutrophils [72,73]. This hyperactive coagulation causes an increase in the level of D-Dimer, a fibrin degradation product [73]. In our study, D-Dimer was significantly higher in non-survivors than in survivors, and there was a positive correlation between D-Dimer and the need for MV and ICU treatment. D-Dimer > 500 µg/L FEU at admission was significantly more frequent in non-survivors compared to survivors and there was also an association of D-Dimer > 500 µg/L FEU at admission and at the 7th DOH and the need for MV and ICU treatment. Moreover, univariate logistic regression revealed that D-Dimer > 500 µg/L FEU at admission and at the 7th DOH were associated with increased risk of death, MV and ICU treatment, and D-Dimer > 500 µg/L FEU at admission was also significantly associated with the risk of ICU treatment in multivariate analysis. This accords with the findings of previous studies of young adults with COVID-19, which have found higher D-Dimer levels in deceased vs. alive patients [31,34]. Conversely, other studies have failed to find associations of this marker with mortality [33] and disease severity [61,79]. Some studies have also found elevated PT [31,34] and APTT [31] in non-survivors, while in a study by Lu et al. [34], APTT did not differ between the non-survivors and the survivors, and in a study by Zhou et al. [79] there was no significant difference in PT between mild and severe cases. In our study, APTT at admission, but not at the 7th DOH, was significantly longer in survivors compared to non-survivors, while there were no significant differences between these groups regarding PT. Moreover, although Zhou et al. found higher fibrinogen in severe vs. mild patients [79], we did not observe this relationship. Furthermore, similar to the findings of Liu et al. [31], we did not find differing AT III levels between survivors and non-survivors. It is worth noting that although pro-inflammatory cytokines may increase platelet production, some authors have also found that severe COVID-19 is characterized by thrombocytopenia, which may result from viral infection of bone marrow and decreased platelet production, or increased platelet consumption due to their abnormal activation by immune complexes or excessive thrombosis [83]. Indeed, in a study of 18–50-year-olds, Zhou et al. [79] found decreased PLT counts in patients with severe compared to mild COVID-19. However, we found no significant difference between non-survivors and survivors regarding PLT counts, which was similar to the results of a study among young adults by Lu et al. [34] One possible explanation is that, as mentioned, severe COVID-19 can cause both thrombocytopenia and increased PLT production. Indeed, we have observed a positive correlation between both thrombophilia and thrombocytopenia and the need for ICU treatment.

Some evidence indicates that lower RBC counts and hemoglobin may be associated with a worse outcome [69,84]. However, other authors have not confirmed this relationship [67,68], including in studies of young adults [34,79]. Interestingly, in our study, although there were no significant differences regarding RBC counts, hematocrit, and hemoglobin levels at admission, at the 7th DOH these parameters were significantly lower in non-survivors than in survivors, and there was a negative correlation between RBC counts, hematocrit and hemoglobin levels at the 7th DOH, and the need for MV and ICU treatment. In addition, univariate logistic regression revealed that RBC count < 4.5 ×10^6^/μL, hematocrit < 40%, and hemoglobin < 12 g/dL at the 7th DOH were associated with increased risk of death, MV and ICU treatment. This accords with a study by Lanser et al. [85] which found a more distinct decrease in hemoglobin levels in patients with severe COVID-19, and the association of new-onset anemia with a higher risk of ICU admission, which the authors suggested reflects hyperinflammation leading to disease progression.

Another biomarker associated with COVID-19 severity is LDH, an enzyme that is present in all tissues and released into the blood upon tissue damage, including such as viral infection, hypoxia, and inflammation-induced injury [86]. In our study, LDH at admission was significantly higher in non-survivors than in survivors, and there was a positive correlation between LDH, and the need for MV and ICU treatment. In addition, LDH > 500 U/L at admission was associated with increased risk of death, MV and ICU treatment in univariate analyses, and with MV and ICU treatment in multivariate analyses. This accords with another study in young adults [31]. However, other authors [33,34] have failed to find this relationship. The elevation of LDH levels may be due to the multiple organ damage, including renal, myocardial, and liver dysfunction, that has been observed in severe COVID-19 [71]. This multiorgan damage is likely multifactorial and may occur either by direct viral invasion through ACE2 receptors expressed in multiple tissues, including myocardium, renal tubular cells and hepatic tissue, or by indirect injury due to cytokine storm and systemic inflammation, sepsis, hypovolemia, hypoxemia, oxidative stress, microvascular thrombosis, and endothelial damage [61,87,88,89,90,91].

According to current evidence, elevation of cardiac injury biomarkers, such as troponin and hs-TnI, CK-MB, myoglobin and NT-proBNP, is associated with COVID-19 severity and mortality [87,88,92]. Agreeing with this evidence, in our study, hs-TnI, CK-MB, myoglobin, and NT-proBNP were significantly higher in non-survivors than in survivors, and there was a positive correlation between hs-TnI, NT-proBNP, and CK-MB and the need for MV and ICU treatment. Moreover, univariate logistic regression revealed that CK-MB > 20 U/L at admission and at the 7th DOH, NT-proBNP > 190 pg/mL at admission and at the 7th DOH, and hsTnI > 34 pg/mL at the 7th DOH were associated with increased risk of death, MV and ICU treatment, and hsTnI > 34 pg/mL at admission—with increased risk of MV and ICU treatment. These findings are consistent with other studies in young adults that found higher levels of hs-TnI [34], CK-MB [31], myoglobin [31,34], and NT-proBNP [31,34] in deceased vs. alive patients, however, there are other studies that did not find differences in the troponin levels between survivors and non-survivors [33], nor differences in CK-MB levels between severe and mild COVID-19 patients [61,79]. We have also observed a correlation between myoglobin levels, a marker that is less specific to cardiac injury, and the need for MV and ICU treatment. Interestingly, another non-cardiac specific marker, CK, was significantly higher in non-survivors than in survivors, but only at the 7th DOH, and not at admission, and in univariate logistic regression CK > 190 U/L at admission and at the 7th DOH were associated with increased risk of MV and ICU treatment, but only CK > 190 U/L at the 7th DOH was associated with the increased risk of death. This might indicate the progression of muscle damage, including rhabdomyolysis and myocardial injury [71,92], in the course of COVID-19. CK was also found to be higher in severe than in mild young adult cases by Zhou et al. [79], but not by Maldonado-Cabrera et al. [61], and Lu et al. [34] did not find a significant difference in CK levels between deceased and alive patients.

Acute kidney injury has also been found to be a predictor of mortality and severity in COVID-19 patients [89,90,91], and higher levels of serum creatinine and blood urea nitrogen (BUN) have also been associated with an increase in fatality and severe disease [89,90]. In our study, creatinine and urea levels were significantly higher in the non-survivors compared to the survivors, and EGFR was significantly lower in non-survivors compared to survivors. We also observed a positive correlation between creatinine levels and the need for MV, a positive correlation between urea levels and the need for MV and ICU treatment, and a negative correlation between EGFR and the need for MV. Furthermore, univariate logistic regression revealed that EGFR < 60 mL/min at admission and at the 7th DOH, creatinine > 1.2 mg/dL at admission and at the 7th DOH and urea > 49 mg/dL at the 7th DOH were associated with increased risk of death, MV and ICU treatment, and urea > 49 mg/dL at admission with increased risk of MV. Other studies in young adults have also found higher creatinine [33,34] and urea [31,34] levels in deceased vs. alive patients. However, creatinine levels did not differ between non-survivors and survivors in a study by Liu et al. [31] nor between mild and severe cases in a study by Zhou et al. [79].

Although COVID-19 has also been hypothesized to cause hepatic injury, data on the association of liver enzyme levels and COVID-19 severity and mortality are inconsistent [93,94,95]. Moreover, hypertransaminasemia observed in some studies may also be due to myocardial and muscle injury or drug-induced hepatotoxicity [93,96]. In our study, ALT at the 7th DOH was significantly higher in survivors compared with non-survivors, however, no other significant differences between these groups regarding ALT, AST, and total bilirubin levels were found. This is in contrast with a study of COVID-19 young adults by Liu et al. [31] that reported ALT, AST, GGT, and bilirubin to be significantly higher in deceased vs. alive patients. Moreover, Zhou et al. [79] found elevated AST in severe vs. mild patients. However, other studies of young adults have found no difference in ALT and AST levels between survivors and non-survivors [33,34], and Zhou et al. [79] did not observe any difference in bilirubin levels between severe vs. mild patients. Interestingly, although there were no significant differences between survivors and non-survivors regarding GGT level, in univariate logistic regression, we found that GGT > 120 U/L at the 7th DOH was associated with increased risk of death, MV, and ICU admission, and GGT > 120 U/L at admission was associated with increased risk of death and MV.

We found albumin concentrations to be significantly lower in non-survivors than in survivors, and there was a moderate to strong negative correlation between albumin concentration and the need for MV and ICU treatment. This agrees with a meta-analysis by Soetedjo et al., which found that hypoalbuminemia was associated with poor prognosis in COVID-19 patients [97]. Similarly, it agrees with previous studies in young adults, which found lower albumin levels in deceased vs. alive patients [31,34], and in severe vs. mild patients [79]. However, decreased albumin levels may not only result from hepatic dysfunction, but also from prioritizing of acute phase proteins synthesis, cytokine-induced increase in vascular permeability leading to albumin extravascular escape, and excessive renal losses due to kidney injury [93,98]. In addition, as albumin has the ability to reduce tissue-damaging oxidative stress and acts as an anticoagulant due to its ability to bind AT III and inhibit platelet aggregation, hypoalbuminemia may further worsen the prognosis [98]. Hence, the prevalence of liver injury in COVID-19 patients may be overestimated [96].

In meta-analyses, hypocalcemia has been found to be significantly associated with COVID-19 severity and mortality [99,100]. In a study by Yang et al. [101], low calcium and phosphorus levels were more prevalent in severe or critical than in moderate COVID-19 patients. The possible mechanisms for a decrease in serum calcium levels in COVID-19 patients include chronic vitamin D deficiency, especially in older patients, hypoalbuminemia, renal insufficiency, the imbalance of parathyroid hormone caused by proinflammatory cytokines, and elevated levels of unsaturated fatty acids that can bind to calcium [99,100]. It is also important to note that calcium is involved in the immune response [99,101]. In our study, total calcium concentration was significantly lower in the non-survivors than in survivors and there was a negative correlation between calcium concentration and the need for MV and ICU treatment. Moreover, univariate logistic regression revealed that calcium < 2.1 mmol/L at admission and at the 7th DOH were associated with increased risk of death, MV and ICU treatment.

Furthermore, vitamin D, an important regulator of calcium homeostasis, has an immunomodulatory role by influencing the production of antimicrobial peptides, as well as counteracting the cytokine storm by inhibiting the production of pro-inflammatory cytokines and promoting anti-inflammatory cytokines, controlling T-cell mediated responses, and modulating the activity of neutrophils and macrophages [102,103,104]. Several meta-analyses found vitamin D deficiency to be associated with a higher risk of developing severe disease, while data regarding its impact on mortality are inconsistent [103,104,105,106]. In our study, we found vitamin D3 levels at admission to be significantly lower in non-survivors than in survivors. Moreover, there was a negative correlation between vitamin D3 levels at the 7th DOH and the need for ICU treatment.

Our study has several limitations. Firstly, it was a single-center retrospective study with a limited sample size that only included severe, hospitalized patients, which may limit the validity of generalizing its results to the entire young adult population. Therefore, larger, multi-center, prospective studies are needed. Secondly, because this study is an observational and exploratory study in which many statistical tests were performed, our results may be influenced by some false-positive error and confounding factors. Moreover, because of the retrospective nature of this study and the limited resources of the health care system at the time, genotypic results of the causative variant were not available. Hence, conclusions regarding the comparison of the wild-type and alpha variants remain presumptive. However, the prevalence of the alpha variant in Poland during the period defined here as the second wave was low (approximately 6.5%), while in the period defined as the third wave the alpha variant accounted for over 92% of the identified strains [10]. These data strongly support that the analyzed waves correspond well to the causative variant.

Therefore, we believe that our study provides valuable data on the impact of infection with the alpha variant of SARS-CoV-2, compared to with wild-type variants, on the severity of the disease, which, in our opinion, might also be of importance in the discussion of the pathogenicity of the next SARS-CoV-2 variants. Noteworthy, none of the patients was vaccinated nor did they have any previous documented SARS-CoV-2 infection. Moreover, there were no significant differences between the waves in terms of the medical treatment used. Hence, we believe that although the variants studied here are no longer dominant, our results are still relevant, as they may provide valuable information on the mechanisms involved in SARS-CoV-2 infection not affected by these factors, which is difficult to achieve in the studies of later variants. Furthermore, most previous studies have focused on predictors of severe COVID-19 in the general population, with older individuals often predominating among those hospitalized, while risk factors among younger individuals appear to be different. In our study, we propose possible predictors of poor COVID-19 outcomes in hospitalized young adults, which may contribute to the early identification of people in this age group at risk of developing severe disease.

## 5. Conclusions

In hospitalized young adults, the SARS-CoV-2 alpha variant does not appear to cause more severe disease than the wild-type variants. Further studies in this age group can be of great use in establishing the influence of current and potential future VOCs on the disease severity. We suggest that a number of factors, including obesity, comorbidities, current or former smoking, the percentage of lung involvement on CT, lower SpO_2_, leukocytosis, neutrophilia, lymphopenia, higher IG count, NLR, and higher CRP, PCT, IL-6, D-Dimer, LDH, hs-TnI, CK-MB, myoglobin, NT-proBNP, creatinine, urea and GGT levels, lower EGFR, albumin, calcium and vitamin D3 levels, and possibly a decrease in RBC counts and hemoglobin and hematocrit levels and an increase in CK levels in the course of hospitalization may be associated with poor outcomes of COVID-19. The earlier identification of young, high-risk patients and appropriate intervention may improve outcomes. As severe disease and deaths also occur in young adults, health authorities should emphasize the need for preventative measures and support research on predictors of poor outcomes in this age group.

## Figures and Tables

**Table 1:** Comparison of general patients’ characteristics between the second and the third wave.

Parameter		Total (*n* = 229)	Second Wave (*n* = 75)	Third Wave (*n* = 154)	*p*-Value
Sex (*n* = 229)	female	57 (24.89%)	16 (21.33%)	41 (26.62%)	0.480 *
	male	172 (75.11%)	59 (78.67%)	113 (73.38%)	
Age, years (*n* = 229)		40 (34–43)	40 (33.5–42)	40 (35–43)	0.392 **
Weight, kg (*n* = 160)		100 (84–110)	100 (90–110)	98 (82–109) (w/o median 97.81)	0.147 ** (w/o 0.178)
BMI, kg/m^2^ (*n* = 154)		30.58 (27.07–34.33)	31.25 (27.45–33.95) (w/o median 31.02)	29.74 (29.6–34.34)	0.316 ** (w/o 0.455)
Comorbidities (*n* = 229)	Comorbidities (any of the following)	93 (40.61%)	35 (46.67%)	58 (37.66%)	0.247 *
	Hypertension	34 (14.85%)	9 (12%)	25 (16.23%)	0.517 *
	Asthma	18 (7.86%)	8 (10.67%)	10 (6.49%)	0.401 *
	Chronic arrhythmia	5 (2.18%)	2 (2.67%)	3 (1.95%)	0.664 ***
	Diabetes	18 (7.86%)	7 (9.33%)	11 (7.14%)	0.752 *
	Insulin resistance	6 (2.62%)	2 (2.67%)	4 (2.6%)	1 ***
	Dyslipidemia	6 (2.62%)	1 (1.33%)	5 (3.25%)	0.667 ***
	Hyperthyroidism	9 (3.93%)	3 (4%)	6 (3.9%)	1 ***
	Hashimoto disease	7 (3.06%)	2 (2.67%)	5 (3.25%)	1 ***

w/o: without outliers; * Chi-squared with Yates correction; ** Mann–Whitney *U* test; *** Fisher’s exact test. Continuous variables are presented as median (IQR), categorical variables are presented as *n* (%).

**Table 2:** Comparison of clinical patients’ characteristics between the second and the third wave.

Parameter		Total (*n* = 229)	Second Wave (*n* = 75)	Third Wave (*n* = 154)	*p*-Value
Symptoms (*n* = 229)	Dyspnea	210 (91.7%)	69 (92%)	141 (91.56%)	1 *
	Fever	195 (85.15%)	66 (88%)	129 (83.77%)	0.517 *
	Cough	195 (85.15%)	62 (82.67%)	133 (86.36%)	0.589 *
	Fatigue	130 (56.77%)	40 (53.33%)	90 (58.44%)	0.555 *
	Diarrhea	41 (17.9%)	9 (12%)	32 (20.78%)	0.149 *
	Nausea or vomiting	27 (11.79%)	10 (13.33%)	17 (11.04%)	0.774 *
	Myalgia	67 (29.26%)	22 (29.33%)	45 (29.22%)	1 *
	Sore throat	18 (7.86%)	2 (2.67%)	16 (10.39%)	0.076 *
	Headache	42 (18.34%)	11 (14.67%)	31 (20.13%)	0.412 *
	Smell and/or taste disorders	43 (18.78%)	18 (24%)	25 (16.23%)	0.218 *
	Smell disorders	37 (16.16%)	16 (21.33%)	21 (13.64%)	0.196 *
	Taste disorders	37 (16.16%)	15 (20%)	22 (14.29%)	0.339 ***
	Hemoptysis	8 (3.49%)	4 (5.33%)	4 (2.6%)	0.444 ***
Percentage of lung involvement on CT,% (*n* = 207)		31 (20.5–45)	30 (20–45)	33 (25–50)	0.319 **
Lung involvement on CT ≥ 50% (*n* = 207)		51 (24.64%)	13 (20.31%)	38 (26.57%)	0.429 *
Death (*n* = 229)		16 (6.99%)	8 (10.67%)	8 (5.19%)	0.212 *
Conventional oxygen therapy (*n* = 229)		222 (96.94%)	73 (97.33%)	149 (96.75%)	1 ***
HFNO (*n* = 229)		55 (24.02%)	20 (26.67%)	35 (22.73%)	0.624 ***
Mechanical ventilation (*n* = 229)		22 (9.61%)	11 (14.67%)	11 (7.14%)	0.115 *
ICU admission (*n* = 229)		31 (13.54%)	15 (20%)	16 (10.39%)	0.074 *
ICU mortality (*n* = 31)		15 (48.39%)	8 (53.33%)	7 (43.75%)	0.862 *

* Chi-squared with Yates correction; ** Mann–Whitney *U* test; *** Fisher’s exact test. Continuous variables are presented as median (IQR), categorical variables are presented as *n* (%).

**Table 3:** Comparison of patients’ laboratory parameters (as continuous variables) between the second and the third wave.

Parameter	Total (*n* = 229)	Second Wave (*n* = 75)	Third Wave (*n* = 154)	*p*-Value *
WBC—at admission, ×10^3^/μL (*n* = 227)	6.39 (4.53–8.31)	7.48 (5.67–11.2) (w/o median 7)	5.66 (4.32–7.41)	<0.001
WBC—7. DOH, ×10^3^/μL (*n* = 187)	8.72 (6.97–10.61)	8.99 (7.11–10.37)	8.68 (6.92–10.64)	0.699 (w/o 0.691)
Neutrophil count—at admission, ×10^3^/μL (*n* = 226)	4.8 (3.17–6.66)	5.82 (3.93–9.2) (w/o median 5.58)	3.98 (2.99–5.92)	<0.001
Neutrophil count—7. DOH, ×10^3^/μL (*n* = 187)	5.5 (4.2–7.36)	5.79 (4.44–7.25)	5.3 (4.11–7.43)	0.479 (w/o 0.341)
Neutrophil percentage—at admission, % (*n* = 226)	77.1 (66.85–83.05)	79.8 (69.7–85.8)	75.7 (65.85–81.05)	0.011 (w/o 0.016)
Neutrophil percentage—7. DOH, % (*n* = 186)	62.7 (54.3–73.28)	63.5 (56.8–71.4)	62.4 (53.9–73.5)	0.574
Lymphocyte count—at admission, ×10^3^/μL (*n* = 226)	0.94 (0.69–1.24)	0.93 (0.68–1.25)	0.94 (0.7–1.24)	0.700 (w/o 0.690)
Lymphocyte count—7. DOH, ×10^3^/μL (*n* = 186)	1.98 (1.31–2.72)	1.87 (1.3–2.65)	2.05 (1.35–2.83) (w/o median 2.04)	0.359 (w/o 0.446)
Lymphocyte percentage—at admission, % (*n* = 226)	15.45 (10.3–23.23)	11.8 (8.1–21.35)	17.2 (12–24.4) (w/o median 17.1)	0.004 (w/o 0.003)
Lymphocyte percentage—7. DOH, % (*n* = 186)	23.95 (14.75–32.28)	21.7 (12.5–30.3)	24.4 (16.1–33.1) (w/o median 24.3)	0.172 (w/o 0.198)
NLR—at admission (*n* = 226)	5.04 (2.85–8.23)	6.59 (3.31–10.25) (w/o median 6.23)	4.36 (2.72–6.69)	0.005 (w/o 0.010)
NLR—7. DOH (*n* = 186)	2.66 (1.7–4.93)	2.88 (1.81–6.38)	2.55 (1.6–4.68)	0.275 (w/o 0.209)
IG count—at admission, ×10^3^/μL (*n* = 217)	0.03 (0.02–0.07)	0.05 (0.03–0.1)	0.03 (0.02–0.05)	0.001
IG count—7. DOH, ×10^3^/μL (*n* = 186)	0.20 (0.09–0.39)	0.24 (0.08–0.41)	0.16 (0.09–0.39)	0.301 (w/o 0.468)
IG percentage—at admission, % (*n* = 217)	0.6 (0.4–0.9)	0.6 (0.5–1.1)	0.5 (0.4–0.8)	0.027 (w/o 0.020)
IG percentage—7. DOH, % (*n* = 186)	2.2 (1.1–4.05)	2.9 (1.1–4.2) (w/o median2.79)	2.1 (1.1–3.9)	0.221 (w/o 0.406)
PLT—at admission, ×10^3^/μL (*n* = 227)	217 (164.5–282.5)	237 (196–307.5) (w/o median 236)	203 (157.75–168.5)	0.009 (w/o 0.011)
PLT—7. DOH, ×10^3^/μL (*n* = 187)	396 (326.5–473.5)	394 (326.25–460.5)	400 (339–477) (w/o median 402)	0.534 (w/o 0.374)
CRP—at admission, mg/L (*n* = 222)	73.2 (35.8–132.5)	87.2 (45–144.95)	63.5 (34.75–121)	0.074 (w/o 0.118)
CRP—7. DOH, mg/L, (*n* = 187)	8.8 (4.2–28.5)	12.7 (5.35–50.7)	7.55 (3.83–19.9)	0.049 (w/o 0.077)
PCT—at admission, ng/mL (*n* = 205)	0.12 (0.07–0.2)	0.14 (0.08–0.23)	0.11 (0.07–0.19)	0.088 (w/o 0.082)
PCT—7. DOH (*n* = 102)	0.08 (0.05–0.17)	0.14 (0.06–0.27)	0.08 (0.05–0.12)	0.023
D-Dimer—at admission, µg/L FEU (*n* = 206)	728 (503–1136.5)	797.5 (566.5–1195.75)	710 (480.5–1118.75)	0.156
D-Dimer—7. DOH, µg/L FEU, *n* (%) (*n* = 150)	867 (539.5–1556.5)	1399 (823–2763)	793 (512–1238)	0.001
PT—at admission, s (*n* = 198)	13.2 (12.3–13.8)	13.4 (12.73–14.68) (w/o 13.35)	13 (11.98–13.7)	0.010 w/o 0.018)
PT—7. DOH, s (*n* = 80)	12.2 (11.7–13.03)	12.5 (12–13.6)	12.1 (11.6–12.7)	0.065 (w/o 0.270)
INR—at admission, s (*n* = 199)	1.19 (1.1–1.25)	1.22 (1.15–1.33)	1.17 (1.08–1.22)	0.003 (w/o 0.006)
INR—7. DOH (*n* = 80)	1.11 (1.06–1.18)	1.12 (1.09–1.22)	1.1 (1.05–1.16)	0.082 (w/o 0.060)
APTT—at admission, s (*n* = 196)	33 (29.78–37.13)	31.7 (28.9–36.4)	33.4 (30.45–37.25)	0.039 (w/o 0.042)
APTT—7. DOH, s (*n* = 65)	32.1 (28.2–36.6)	32.5 (28.5–36.4)	31.95 (27.78–38.7)	0.944
Albumin—at admission, g/dL (*n* = 26)	3.34 (3.18–3.65)	3.22 (3.02–3.65)	3.42 (3.27–3.57)	0.315
Albumin—7. DOH, g/dL (*n* = 19)	3.03 (2.74–3.38)	2.77 (2.65–2.98)	3.38 (3.11–3.42)	0.007

* Mann–Whitney *U* test; w/o: without outliers. All variables are presented as median (IQR).

**Table 4:** Comparison of general patients’ characteristics between survivors and non-survivors.

Parameter		Survivors (*n* = 213)	Non-Survivors (*n* = 16)	*p*-Value
Sex (*n* = 229)	female	51 (23.94%)	6 (37.5%)	0.236 *
	male	162 (76.06%)	10 (62.5%)	
Age, years (*n* = 229)		40 (34–43)	41.5 (39.5–43)	0.152 **
Weight, kg (*n* = 160)		98 (83–110) (w/o median 97.81)	105 (98–123.5) (w/o median 104)	0.026 ** (w/o 0.063)
BMI, kg/m^2^ (*n* = 154)		29.74 (26.46–33.95)	34.26 (29.59–38.73) (w/o median 33.06)	0.019 ** (w/o 0.097)
BMI, ranges (*n* = 154)	<25	18 (12.95%)	1 (6.67%)	0.696 *
	25–29.9	52 (37.41%)	3 (20%)	0.292 *
	30–34.9	41 (29.5%)	5 (33.33%)	0.771 *
	35–39.9	20 (14.39%)	3 (20%)	0.472 *
	≥40	8 (5.76%)	3 (20%)	0.077 ***
Comorbidities (*n* = 229)	Comorbidities (any of the following)	82 (38.5%)	11 (68.75%)	0.035 *
	Hypertension	30 (14.08%)	4 (25%)	0.268 *
	Asthma	17 (7.98%)	1 (6.25%)	1 *
	Chronic arrhythmia	3 (1.41%)	2 (12.5%)	0.041 ***
	Diabetes	15 (7.04%)	3 (18.75%)	0.199 *
	Insulin resistance	4 (1.88%)	2 (12.5%)	0.058 ***
	Dyslipidemia	5 (2.35%)	1 (6.25%)	0.356 ***
	Hypothyroidism	8 (3.76%)	1 (6.25%)	0.485 ***
	Hashimoto disease	7 (3.29%)	0 (0%)	1 ***
Smoking (*n* = 164)	Current	11 (7.14%)	2 (20%)	0.182 ***
	Current or former	16 (10.39%)	4 (40%)	0.021 *
Blood type (*n* = 62)	A Rh+	12 (25%)	6 (42.86%)	0.315 *
	A Rh−	3(6.25%)	0 (0%)	1 ***
	B Rh+	11 (22.92%)	1 (7.14%)	0.267 *
	B Rh−	2 (4.17%)	2 (14.29%)	0.217 ***
	AB Rh+	2 (4.17%)	0 (0%)	1 ***
	AB Rh−	2 (4.17%)	0 (0%)	1 ***
	0 Rh+	15 (31.25%)	5 (35.71%)	0.755 *
	0 Rh−	1 (2.08%)	0 (0%)	1 ***

w/o: without outliers; * Chi-squared with Yates correction; ** Mann–Whitney *U* test; *** Fisher’s exact test. Continuous variables are presented as median (IQR), categorical variables are presented as *n* (%).

**Table 5:** Comparison of clinical patients’ characteristics between survivors and non-survivors.

Parameter	Survivors (*n* = 213)	Non-Survivors (*n* = 16)	*p*-Value
Percentage of lung involvement on CT, % (*n* = 207)	30 (20–44.25)	70 (40–85)	<0.001 **
Lung involvement on CT ≥ 50% (*n* = 207)	42 (21.65%)	9 (69.23%)	0.001 *
SpO_2_ at admission, % (*n* = 190)	90 (87–92)	85 (70–89)	0.004 **
Time from the onset of symptoms to hospital admission, days	8 (7–11)	7 (5.5–9)	0.048 **
Conventional oxygen therapy (*n* = 229)	206 (96.71%)	16 (100%)	1 ***
Maximum oxygen flow (conventional oxygen therapy), l/min (*n* = 214)	6.5 (5–15)	15 (15–15)	<0.001 **
HFNO (*n* = 229)	41 (19.25%)	14 (87.5%)	<0.001 ***
Maximum flow—HFNO (l/min; (*n* = 55)	60 (55–60)	60 (60–78.75)	0.003 **
Maximum FiO2—HFNO,% (*n* = 55)	90 (78–95)	95 (90.5–98.25)	0.014 **
Mechanical ventilation (*n* = 229)	6 (2.82%)	16 (100%)	<0.001 *
Extubation; (*n* = 22)	6 (100%)	0 (0%)	<0.001 ***
ECMO (*n* = 229)	6 (2.82%)	2 (12.5%)	0.100 ***
ICU admission (*n* = 229)	16 (7.51%)	15 (93.75%)	<0.001 *
Time from the onset of symptoms to ICU admission, days	10 (8.75–13)	10 (8.5–13)	1 **
Vasopressors (*n* = 229)	6 (2.82%)	15 (93.75%)	<0.001 *
CRRT (*n* = 229)	0 (0%)	5 (31.25%)	<0.001 ***

* Chi-squared with Yates correction; ** Mann–Whitney *U* test; *** Fisher’s exact test. Continuous variables are presented as median (IQR), categorical variables are presented as *n* (%).

**Table 6:** Comparison of patients’ laboratory parameters (as continuous variables) between survivors and non-survivors.

Parameter	Survivors (*n* = 213)	Non-Survivors (*n* = 16)	*p*-Value *
**Inflammatory Markers**			
WBC—at admission, ×10^3^/μL (*n* = 227)	6.12 (4.5–8.1)	9.43 (6.95–12.99)	0.001
WBC—7. DOH, ×10^3^/μL (*n* = 187)	8.66 (6.84–10.2)	13.33 (10.1–20.67)	<0.001
Neutrophil count—at admission, ×10^3^/μL (*n* = 226)	4.46 (3.12–6.39)	7.69 (5.21–11.58)	0.001
Neutrophil count—7. DOH, ×10^3^/μL (*n* = 187)	5.35 (4.11–6.99)	11.69 (7.73–13.64)	<0.001
Neutrophil percentage—at admission, *n*(%) (*n* = 226)	76.9 (66.33–82)	82.65 (76.88–87.28)	0.007
Neutrophil percentage—7. DOH,% (*n* = 186)	61.9 (53.9–70.3)	84.5 (74.2–86.2)	<0.001
Lymphocyte count—at admission, ×10^3^/μL (*n* = 226)	0.93 (0.69–1.24)	0.97 (0.72–1.25)	0.896
Lymphocyte count—7. DOH, ×10^3^/μL (*n* = 186)	2.07 (1.37–2.79)	0.9 (0.76–1.49)	<0.001
Lymphocyte percentage—at admission,% (*n* = 226)	16.7 (10.4–23.95)	11.35 (7.38–12.3)	0.001
Lymphocyte percentage—7. DOH, % (*n* = 186)	24.4 (17.5–32.8)	6.9 (6.4–9)	<0.001
NLR—at admission (*n* = 226)	4.49 (2.77–7.83)	7.11 (6.52–11.33)	0.002
NLR—7. DOH (*n* = 186)	2.52 (1.66–4.16)	11.58 (7.42–13.1)	<0.001
IG count—at admission, ×10^3^/μL (*n* = 217)	0.03 (0.02–0.06)	0.09 (0.06–0.14)	<0.001
IG count—7. DOH, ×10^3^/μL (*n* = 186)	0.18 (0.08–0.37)	0.64 (0.33–0.76)	0.001
IG percentage—at admission,% (*n* = 217)	0.5 (0.4–0.8)	1.05 (0.7–1.3)	<0.001
IG percentage—7. DOH,% (*n* = 186)	2.2 (1–3.8)	4.9 (2.6–9.1)	0.009
CRP—at admission, mg/L (*n* = 222)	70.5 (34.65–126.4)	145.6 (82.11–161.7)	0.006
CRP—7. DOH, mg/L, (*n* = 187)	7.6 (3.85–18.6)	91.75 (55.73–127.75)	<0.001
PCT—at admission, ng/mL (*n* = 205)	0.11 (0.07–0.18)	0.39 (0.18–1.22)	<0.001
PCT—7. DOH, ng/mL (*n* = 102)	0.07 (0.05–0.13)	0.61 (0.29–0.74)	<0.001
Ferritin—at admission, ng/mL (*n* = 61)	1156 (473–1498)	2128 (1604–2716.75)	0.088
Ferritin—7. DOH, ng/mL (*n* = 31)	901.5 (430–1343.25)	1455 (1455–1455)	–
IL-6—at admission, ng/mL (*n* = 116)	18.5 (6.72–53.78)	106.4 (69.43–160.75)	0.015
IL-6—7. DOH, ng/mL (*n* = 53)	7.28 (4.01–19.1)	65.7 (24.35–741.5)	0.041
AT III—at admission,% (*n* = 17)	94.5 (85–100.25)	95 (80.5–117)	0.732
AT III—7. DOH,% (*n* = 10)	112 (101.5–117.5)	91 (84.5–95.5)	0.252
**Coagulation Parameters**			
PLT—at admission, ×10^3^/μL (*n* = 227)	217 (161–280.5)	225 (194–322.75)	0.253
PLT—7. DOH, ×10^3^/μL (*n* = 187)	398 (339.25–473.75)	353 (240–471)	0.334
D-Dimer—at admission, µg/L FEU (*n* = 206)	712 (487–1123)	991 (728–1593.5)	0.015
D-Dimer—7. DOH, µg/L FEU (*n* = 150)	823 (531–1363.25)	1810.5 (1346.75–5268)	<0.001
PT—at admission, s (*n* = 198)	13.2 (12.3–13.8)	12.9 (11.98–14)	0.767
PT—7. DOH, s (*n* = 80)	12.2 (11.7–13.1)	12.5 (12.05–13)	0.695
INR—at admission (*n* = 199)	1.19 (1.11–1.25)	1.14 (1.07–1.27)	0.541
INR—7. DOH (*n* = 80)	1.11 (1.06–1.19)	1.12 (1.1–1.17)	0.883
APTT—at admission, s (*n* = 196)	33.25 (30.2–37.35)	29.25 (27.28–32.45)	0.012
APTT—7. DOH, s (*n* = 65)	32.1 (28.6–35.7)	34.1 (25.88–46.73)	0.750
Fibrinogen—at admission, mg/dL (*n* = 62)	598 (501.75–737.75)	531 (392.5–673.75)	0.255
Fibrinogen—7. DOH, mg/dL (*n* = 39)	481.5 (385.5–553.5)	613 (410–702)	0.279
RED BLOOD CELL INDICES			
RBC count—at admission, ×10^6^/μL (*n* = 227)	4.78 (4.53–5.05)	4.95 (4.7–5.08)	0.269
RBC count—7. DOH, ×10^6^/μL (*n* = 187)	4.77 (4.5–5.06)	3.85 (3.43–4.12)	<0.001
Hemoglobin—at admission, g/dL (*n* = 227_	14.3 (13.5–15.1)	14.55 (13.15–15.35)	0.699
Hemoglobin—7. DOH, g/dL (*n* = 187)	14.2 (13.2–15.18)	11.6 (9.4–12.1)	<0.001
Hematocrit—at admission,% (*n* = 227)	41.5 (39.35–44)	43.45 (39.68–44.13)	0.292
Hematocrit—7. DOH,% (*n* = 187)	41.95 (39.4–44)	35 (30–36.6)	<0.001
**Non-Specific Tissue Damage and Cardiac Injury Markers**			
LDH—at admission, U/L (*n* = 172)	396 (310–533)	783 (591–1257)	<0.001
LDH—7. DOH, U/L (*n* = 46)	387 (250–442)	865 (865–865)	–
Myoglobin—at admission, ng/mL (*n* = 15)	60 (49–118)	2687.5 (721.75–3087.25)	0.039
Myoglobin—7. DOH, ng/mL (*n* = 11)	28 (23.5–76.5)	258.5 (124–300)	0.085
CK—at admission, U/L (*n* = 142)	240 (114–422.25)	426 (97.25–1860.25)	0.236
CK—7. DOH, U/L (*n* = 38)	42.5 (26–101.5)	203.5 (195–261)	0.004
CK-MB—at admission, U/L (*n* = 124)	18 (15–24)	23 (18.85.36.5)	0.022
CK-MB—7. DOH, U/L (*n* = 27)	14 (12–30)	22 (18–30.75)	0.159
NT-proBNP—at admission, pg/mL (*n* = 142)	98 (40.5–175.5)	322 (92–1068)	0.012
NT-proBNP—7. DOH, pg/mL (*n* = 42)	111.5 (47.75–207.75)	429 (205.75–3615.5)	0.001
hs-TnI—at admission, pg/mL (*n* = 153)	3.25 (3.2–6.38)	12.5 (47–23.9)	0.001
hs-TnI—7. DOH, pg/mL (*n* = 40)	3.2 (1.2–3.2)	26.7 (17–165.15)	0.003
**Renal Injury Markers**			
Creatinine—at admission, mg/dL (*n* = 222)	0.9 (0.76–1.03)	0.99 (0.84–1.27)	0.036
Creatinine—7. DOH, mg/dL (*n* = 169)	0.81 (0.72–0.91)	0.88 (0.55–1.96)	0.616
EGFR—at admission, mL/min (*n* = 215)	91 (78–103.5)	79.5 (52.75–91.75)	0.011
EGFR—7. DOH, mL/min (*n* = 169)	102 (89–117.5)	98 (43.5–139.25)	0.572
Urea—at admission, mg/dL (*n* = 209)	27 (21.25–34.75)	37 (27.85–48.5)	0.011
Urea—7. DOH, mg/dL (*n* = 124)	32 (27–37)	52 (42–81.75)	<0.001
**Liver Injury Markers**			
ALT—at admission, U/L (*n* = 226)	47 (33–68)	49.5 (27–88.75)	0.758
ALT—7. DOH, U/L (*n* = 150)	100 (62–154)	42 (28.5–47.5)	0.001
AST—at admission, U/L (*n* = 219)	48 (34.5–70)	53 (39.75–145.5)	0.185
AST—7. DOH, U/L (*n* = 148)	46 (30–71)	37 (25–44)	0.149
GGT—at admission, U/L (*n* = 58)	58 (36.5–137.5)	136 (71–250.5)	0.076
GGT—7. DOH, U/L (*n* = 41)	94 (58–191.75)	158 (105–184)	0.690
Total bilirubin—at admission, mg/dL (*n* = 165)	0.43 (0.31–0.54)	0.58 (0.37–0.99)	0.055
Total bilirubin—7. DOH, mg/dL (*n* = 63)	0.44 (0.31–0.6)	0.3 (0.25–0.52)	0.211
**Other Laboratory Parameters**			
Albumin—at admission, g/dL (*n* = 26)	3.46 (3.15–3.82)	3.22 (3.19–3.28)	0.211
Albumin—7. DOH, g/dL (*n* = 19)	3.36 (3.06–3.44)	2.74 (2.67–2.9)	0.004
Total calcium—at admission, mmol/L (*n* = 42)	2.16 (2.06–2.24)	2.05 (2.02–2.09)	0.049
Total calcium—7. DOH, mmol/L (*n* = 34)	2.27 (2.2–2.32)	2.08 (1.99–2.17)	<0.001
Vitamin D3—at admission, ng/mL (*n* = 81)	27.7 (19.85–33.85)	18.75 (15.38–25.13)	0.173
Vitamin D3—7. DOH, ng/mL (*n* = 6)	22.45 (16.7–35.4)	–	–

w/o: without outliers; * Mann–Whitney *U* test. All variables are presented as median (IQR).

**Table 7:** Univariate logistic regression analysis of selected laboratory parameters at the 7th DOH for the prediction of death, mechanical ventilation, and ICU treatment.

Variable	Death			MV			ICU Treatment		
	OR	95% CI	*p*	OR	95% CI	*p*	OR	95% CI	*p*
CK-MB > 20—7. DOH	26.83	7.47–96.23	<0.001	23.09	6.67–79.9	<0.001	19.84	5.64–69.78	<0.001
CK > 190—7. DOH	70	15.57–314.86	<0.001	70.96	13.88–362.73	<0.001	40.09	8.14–197.45	<0.001
D-Dimer > 500 µg/L FEU—7. DOH	6.94	1.54–31.26	0.012	10.5	2.39–46.05	0.002	10.54	3.1–35.8	<0.001
EGFR < 60 mL/min—7. DOH	35.17	5.85–211.54	<0.001	60.59	6.69–548.62	<0.001	37.89	4.26–337.05	0.001
GGT > 120 U/L—7. DOH	6.99	2.11–23.14	0.001	6.09	2.02–18.39	0.001	4.96	1.76–14.01	0.003
Hematocrit < 40%—7. DOH	6.81	2.26–20.51	<0.001	9.08	3.36–24.51	<0.001	5.54	2.5–12.29	<0.001
Hemoglobin < 12 g/dL—7. DOH	14.21	4.64–43.56	<0.001	17.82	6.34–50.07	<0.001	11.87	4.53–31.1	<0.001
Creatinine > 1.2 mg/dL—7. DOH	23.33	4.68–116.27	<0.001	30.15	5.44–167.16	<0.001	18.85	3.48–102.15	<0.001
Urea > 49—7. DOH	25.6	7.66–85.76	<0.001	179.38	34.75–925.99	<0.001	80.71	16.92–384.95	<0.001
NT-proBNP > 190—7. DOH	15.79	4.88–51.09	<0.001	12.57	4.2–37.61	<0.001	7.3	2.57–20.79	<0.001
RBC < 4.5 × 10^6^/μL—7. DOH	8.7	2.87–26.36	<0.001	11.86	4.35–32.31	<0.001	5.28	2.39–11.67	<0.001
hsTnI > 34 pg/mL—7. DOH	31.82	6.72–150.58	<0.001	38.44	7.17–206.09	<0.001	57.46	6.78–487.27	<0.001
Total calcium < 2.1 mmol/L—7. DOH	31.35	7.61–129.1	<0.001	31.73	7.44–135.37	<0.001	34.09	6.82–170.24	<0.001
NLR ≥ 2—7. DOH	4.46	1.24–16.09	0.023	6.91	1.98–24.06	0.002	11.2	3.3–38.06	<0.001
Lymphocyte count < 0.9 × 10^3^/μL—7. DOH	9.23	2.9–29.36	<0.001	7.58	2.6–22.11	<0.001	5.91	2.16–16.2	<0.001
Lymphocyte percentage < 19%—7. DOH	13.76	3.77–50.22	<0.001	11.57	4.06–33.04	<0.001	13.54	5.45–33.67	<0.001
Neutrophil count > 7 × 10^3^/μL —7. DOH	12.21	3.75–39.79	<0.001	11.86	4.35–32.31	<0.001	7.37	3.28–16.57	<0.001
Neutrophil percentage > 68%—7. DOH	6.32	2.1–19	0.001	5.22	2.07–13.14	<0.001	7.14	3.14–16.26	<0.001
WBC count > 10 × 10^3^/μL—7. DOH	5.73	1.98–16.57	0.001	5.2	2.09–12.94	<0.001	3.92	1.81–8.66	<0.001
CRP ≥ 100 mg/L—7. DOH	42, 95	9.15–192.85	<0.001	47.83	9.13–250.73	<0.001	28.58	5.61–145.54	<0.001
PCT > 0.5 ng/mL—7. DOH	54.44	12.05–246	<0.001	31.73	7.44–135.37	<0.001	34.09	6.82–170.29	<0.001

**Table 8:** Univariate and multivariate logistic regression analysis of selected clinical characteristics and laboratory parameters at admission for the prediction of death.

Variable	Univariate Regression			Multivariate Regression		
	OR	95% CI	*p*	OR	95% CI	*p*
Weight > 100 kg	6.02	2.01–18.09	0.001			
BMI ≥ 40 kg/m^2^	5.91	1.4–27.97	0.016			
Comorbidities	3.51	1.18–10.48	0.024	3.96	1.21–12.98	0.023
Diabetes or insulin resistance	4.6	1.46–14.77	0.009			
Chronic arrhythmia	10	1.54–64.83	0.016			
CK-MB > 20—at admission	3.08	1.08–8.73	0.035			
D-Dimer > 500 µg/L FEU—at admission	7.99	1.03–61.65	0.046			
EGFR < 60 mL/min—at admission	9.23	2.69–31.67	<0.001			
GGT > 120 U/L—at admission	7.92	2.53–24.77	<0.001			
Creatinine > 1.2 mg/dL—at admission	4.92	1.54–15.74	0.007			
LDH > 500 U/L—at admission	4.8	1.7–13.6	0.003			
NT-proBNP > 190 pg/mL—at admission	3.66	1.24–10.81	0.019			
Total calcium < 2.1 mmol/L—at admission	8.35	2.47–28.24	<0.001			
Neutrophil count > 7 × 10^3^/μL—at admission	6.59	2.27–19.13	<0.001			
WBC count > 10 × 10^3^/μL—at admission	7.19	2.47–20.81	<0.001	5.8	1.45–16.95	0.003
CRP ≥ 100 mg/L—at admission	3.26	1.14–9.34	0.027			
PCT > 0.5 ng/mL—at admission	7.39	2.38–22.94	<0.001	4.96	1.45–16.95	0.011

**Table 9:** Univariate and multivariate logistic regression analysis of selected clinical characteristics and laboratory parameters at admission for the prediction of mechanical ventilation.

Variable	Univariate Regression			Multivariate Regression		
	OR	95% CI	*p*	OR	95% CI	*p*
Weight > 100 kg	4.96	1.97–12.47	<0.001			
BMI ≥ 40 kg/m^2^	6.35	1.7–23.76	0.006	6.88	1.27–37.45	0.026
Comorbidities	2.84	1.14–7.06	0.025			
Diabetes or insulin resistance	5.22	1.87–14.54	0.002			
Chronic arrythmia	6.8	1.07–43.13	0.042			
CK-MB > 20 at admission	3.48	1.4–8.62	0.007			
CK > 190 at admission	2.49	1.02–6.1	0.046			
D-Dimer > 500 µg/L FEU—at admission	11.68	1.54–88.62	0.017			
EGFR < 60—mL/min at admission	16.33	5.17–51.57	<0.001			
GGT > 120 U/L—at admission	8.53	3.03–23.99	<0.001			
Creatinine > 1.2 mg/dL—at admission	7.31	2.65–20.19	<0.001			
LDH > 500 U/L—at admission	5.85	2.34–14.62	<0.001	4.67	1.58–13.84	0.005
Urea > 49 at admission	5.24	1.63–16.84	0.005			
NT-proBNP > 190 pg/mL—at admission	5.8	2.28–14.77	<0.001			
hsTnI > 34 pg/mL—at admission	14.93	3.66–60.82	<0.001			
Total calcium < 2.1 mmol/L—at admission	10.27	3.36–31.42	<0.001			
Neutrophil count > 7 × 10^3^/μL—at admission	7.54	2.96–19.22	<0.001			
WBC count > 10 × 10^3^/μL—at admission	10.09	3.91–26.05	<0.001	5.75	1.9–17.37	0.002
CRP ≥ 100 mg/L—at admission	5.7	2.13–15.22	<0.001			
PCT > 0.5 ng/mL—at admission	13.54	4.87–37.62	<0.001	9.56	2.97–30.92	<0.001

**Table 10:** Univariate and multivariate logistic regression analysis of selected clinical characteristics and laboratory parameters at admission for the prediction of ICU treatment.

Variable	Univariate Regression			Multivariate Regression		
	OR	95% CI	*p*	OR	95% CI	*p*
Weight > 100 kg	2.57	1.19–5.55	0.017			
BMI ≥ 40 kg/m^2^	4.04	1.11–14.73	0.034			
Diabetes or insulin resistance	4.99	1.96–12.74	<0.001			
CK-MB > 20 at admission	3.14	1.42–6.98	0.0049			
CK > 190 at admission	2.9	1.33–6.31	0.008			
D-Dimer > 500 µg/L FEU—at admission	8.47	1.96–36.53	0.004	5.24	1.15–23.84	0.032
EGFR < 60 mL/min—at admission	9.49	3.15–28.59	<0.001			
GGT > 120 U/L—at admission	4.95	1.86–13.2	0.001			
Creatinine > 1.2 mg/dL—at admission	3.32	1.24–8.88	0.017			
LDH > 500 U/L—at admission	5.28	2.39–11.67	<0.001	3.35	1.82–16.17	0.002
NT-proBNP > 190 pg/mL—at admission	5.5	2.38–12.67	<0.001			
hsTnI > 34 pg/mL—at admission	9.33	2.35–36.97	0.002			
Total calcium < 2.1 mmol/L—at admission	6.13	2.09–17.95	0.001			
Lymphocyte percentage < 19% at admission	25	3.43–186.95	0.002			
Neutrophil count > 7 × 10^3^/μL—at admission	7.64	3.39–17.2	<0.001			
Neutrophil percentage > 68% at admission	14.66	1.96–109.9	0.009			
WBC count > 10 × 10^3^/μL—at admission	6.09	2.62–14.17	<0.001	3.69	1.38–9.85	0.009
CRP ≥ 100 mg/L—at admission	4.72	2.1–10.62	<0.001			
PCT > 0.5 ng/mL—at admission	9.35	3.6–24.29	<0.001	5.42	1.82–16.17	0.002

## Data Availability

The original, anonymous dataset is available upon request from the corresponding author.

## Supplementary Materials

The following supporting information can be downloaded at: https://www.mdpi.com/article/10.3390/v14081700/s1, Table S1: Additional data on general and clinical patients’ characteristics between the second and the third wave; Table S2: Additional data on comparison of patients’ laboratory parameters (as continuous variables) between the second and the third wave; Table S3: Comparison of patients’ laboratory parameters (as categorical variables) between the second and the third wave; Table S4: Additional data on comparison of general patients’ characteristics between survivors and non-survivors; Table S5: The correlations between patients’ general and clinical characteristics (categorical variables) and the need for mechanical ventilation and ICU treatment; Table S6: The correlations between patients’ general and clinical characteristics and laboratory parameters (presented as continuous variables) and the need for mechanical ventilation and ICU treatment; Table S7: The correlations between patients’ laboratory parameters (presented as categorical variables) and the need for mechanical ventilation and ICU treatment; Table S8: Univariate logistic regression analysis of COVID-19 symptoms for the prediction of death, mechanical ventilation and ICU treatment.
