# Supplementary material for: COVID-19 Severity and Mortality in Two Pandemic Waves in Poland and Predictors of Poor Outcomes of SARS-CoV-2 Infection in Hospitalized Young Adults
Source: Viruses. 2022 Jul 31;14(8):1700. doi: 10.3390/v14081700 (PMC9413321; doi:10.3390/v14081700)
Supplement: Supplementary file 1 [file viruses-14-01700-s001.zip › viruses-1831110-supplementary.pdf]

Table S1. Additional data on general and clinical patients' characteristics between the second and the third wave.

Parameter		Total (N=229)	Second wave (N=75)	Third wave (N=154)	p-value
BMI, ranges (N=154)	18-24.9 kg/m^2	19 (12.34%)	5 (10.2%)	14 (13.33%)	0.774*
	25-29.9 kg/m^2	55 (35.71%)	16 (32.65%)	39 (37.14%)	0.718*
	30-34.9 kg/m^2	46 (29.87%)	16 (32.65%)	30 (28.57%)	0.744*
	35-39.9 kg/m^2	23 (14.94%)	6 (12.24%)	17 (16.19%)	0.691*
	≥ 40 kg/m^2	11 (7.14%)	6 (12.24%)	5 (4.76%)	0.105***
Smoking (N=164)	Current	13 (7.93%)	3 (5.26%)	10 (9.35%)	0.545***
	Current or former	20 (12.2%)	6 (10.53%)	14 (13.08%)	0.821*
Blood type (N=62)	A Rh+	18 (29.03%)	11 (30.56%)	7 (26.92%)	0.978*
	A Rh-	3 (4.84%)	2 (5.56%)	1 (3.85%)	1***
	B Rh+	12 (19.35%)	8 (22.22%)	4 (15.38%)	0.729*
	B Rh-	4 (6.45%)	3 (8.33%)	1 (3.85%)	0.633***
	AB Rh+	2 (3.23%)	1 (2.78%)	1 (3.85%)	1***
	AB Rh-	2 (3.23%)	1 (2.78%)	1 (3.85%)	1***
	0 Rh+	20 (32.26%)	9 (25%)	11 (42.31%)	0.245*
	0 Rh-	1 (1.61%)	1 (2.78%)	0 (0%)	1***
Treatment	Steroids	222 (96.9%)	74 (98.7%)	148 (96.1%)	0.431***
	Remdesivir	59 (25.8%)	19 (25.3%)	40 (26%)	0.955*
	Tocilizumab	9 (3.9%)	3 (4%)	6 (3.9%)	1***
	Antibiotics	166 (72.5%)	56 (74.7%)	110 (71.4%)	0.721*
	Convalescent plasma	20 (8.7%)	7 (9.3%)	13 (8.4%)	0.980*
	LMWH	227 (99.1%)	75 (100%)	152 (98.7%)	0.815*
SpO2 at admission,% (N=190)		90 (87-92)	89 (85.5-92)	90 (87-92)	0.641**
Time from the onset of symptoms to hospital admission, days (N=229)		8 (7-11)	8 (6-10)	9 (7-11)	0.074**
Duration of hospitalization (excluding deceased), days (N=212)		10.5 (8-14)	11 (9-16)	10 (8-13.25)	0.036**
Time from hospital admission to death (N=16)		15 (8.75-27.25)	20 (14.5-24.75)	10 (7.25-30.75)	0.329**
Conventional oxygen therapy (N=229)		222 (96.94%)	73 (97.33%)	149 (96.75%)	1***
Maximum flow – HFNO, l/min (N=55)		60 (57.5-60)	60 (55-61.25)	60 (60-60)	0.814**
Maximum FiO2 – HFNO, % (N=55)		90 (83.5-95)	94.5 (87.5-95.25)	90 (83.5-95)	0.277**
Extubation (N=22)		6 (27.27%)	3 (27.27%)	3 (27.27%)	1***
Duration of oxygen therapy (conventional/HFNO/invasive ventilation), days (N=206)			8 (5-12)	6 (4-10)	0.814**
ECMO (N=229)		8 (3.49%)	4 (5.33%)	4 (2.6%)	0.444***
Length of ICU stay (excluding deceased), days (N=16)		9 (6-18.25)	9 (6-25.5)	9 (6-16)	1**
Time from the onset of symptoms to ICU admission, days (N=31)		10 (8.5-13)	10 (8-13)	10 (9-13)	0.524**
Vasopressors (N=229)		21 (9.17%)	11 (14.67%)	10 (6.49%)	0.077*
CRRT (N=229)		5 (2.18%)	3 (4%)	2 (1.3%)	0.334**

w/o – without outliers; * - Chi-squared with Yates correction; ** - Mann-Whitney U test; *** - Fisher's exact test. Continuous variables are presented as median (IQR), categorical variables are presented as N (%).

LMWH – Low molecular-weight heparin, CRRT - continuous renal replacement therapy.

Table S2. Additional data on comparison of patients' laboratory parameters (as continuous variables) between the second and the third wave.

Parameter	Total (N=229)	Second wave (N=75)	Third wave (N=154)	p-value*
RBC count - at admission, ×10⁶/µL (N=227)	4.79 (4.54-5.05)	4.78 (4.46-5.06) (w/o median 4.8)	4.79 (4.59-5.05)	0.761 (w/o 0.835)
RBC count - 7. DOH, ×10⁶/µL (N=187)	4.72 (4.45-5.04)	4.62 (4.37-4.99) (w/o median 4.67)	4.75 (4.5-5.09)	0.246 (w/o 0.321)
Hemoglobin - at admission, g/dL (N=227)	14.3 (13.45-15.1)	14.3 (13.35-15.25)	14.35 (13.6-15.03)	0.818 (w/o 0.813)
Hemoglobin – 7. DOH, g/dL (N=187)	14 (13-15)	13.95 (12.8-14.8)	14.1 (13.2-15.2)	0.239
Hematocrit - at admission, % (N=227)	41.6 (39.35-44.05)	41.2 (39.2-44)	41.7 (39.68-44.03)	0.375 (w/o 0.417)
Hematocrit – 7. DOH, % (N=187)	41.7 (38.5-44.6)	41.25 (37.3-44.25)	41.9 (39.4-44.6)	0.094 (w/o 0.128)
Ferritin - at admission, ng/mL (N=61)	1160 (487-1562)	1160 (725.5-1386)	1159 (470.5-1567.25)	0.802 (w/o 0.947)
Ferritin – 7. DOH, ng/mL (N=31)	951 (438-1394)	920 (585.75-1484.5)	951 (438-1323.5)	0.740
IL-6 - at admission, ng/mL (N=116)	20.75 (7.2-56.6)	18.8 (8.22-45)	20.8 (5.56-65.2)	0.622
IL-6 – 7. DOH, ng/mL (N=53)	9.1 (4.23-21.8)	9.19 (6.11-22.2)	8.45 (2.34-21.05)	0.426
AT III - at admission, % (N=17)	95 (85-101)	93 (76.5-97)	102.5 (95-128)	0.159
AT III – 7. DOH, % (N=10)	93 (90.25-108)	91 (82-94.75)	109 (93.75-123)	0.238
Fibrinogen - at admission, mg/dL (N=62)	598 (467-728.25)	646 (510-773)	585 (466-683)	0.278 (w/o 0.191)
Fibrinogen – 7. DOH, mg/dL (N=39)	486 (386-578)	532.5 (385.5-619.75)	477 (410-557)	0.548
LDH - at admission, U/L (N=172)	410 (313-554)	380 (292.25-530.75)	419.5 (324.75-575)	0.178
LDH – 7. DOH, U/L (N=46)	389.5 (256.75-447.25)	397 (353-529.75)	368.5 (243.5-434.5)	0.181
hs-TnI - at admission, pg/mL (N=153)	3.4 (3.2-7.9)	3.9 (2.55-7.35)	3.2 (3.2-7.83)	0.667
hs-TnI – 7. DOH, pg/mL (N=40)	3.2 (2.28-22.1)	2.45 (1.08-23.1)	3.2 (3.2-13.25)	0.244
CK - at admission, U/L (N=142)	250.5 (114-457)	207.5 (88.5-465.75)	256.5 (134-452.25)	0.268
CK – 7. DOH, U/L (N=38)	69.5 (35.25-197)	83.5 (25.75-225)	67.5 (36.75-129.25)	0.745
CK-MB - at admission, U/L (N=124)	19 (15-25)	19 (15-22)	18 (15-27)	0.998
CK-MB – 7. DOH, U/L (N=27)	18 (13.5-30.5)	20 (13.5-30)	15 (14-36)	0.865
NT-proBNP - at admission, pg/mL (N=142)	101.5 (43-192.75)	116.5 (49-234.75)	100.6 (40.75-178.25)	0.524
NT-proBNP – 7. DOH, pg/mL (N=42)	147 (57.75-264.25)	208.5 (66.5-272)	119 (65.25-218.25)	0.247
Myoglobin - at admission, ng/mL (N=15)	118 (54.5-1459)	60 (48-218)	1459 (430.75-2579.5)	0.078
Myoglobin – 7. DOH, ng/mL (N=11)	150 (37-277)	125 (28-248)	811 (578-1044)	0.034
Creatinine - at admission, mg/dL (N=222)	0.9 (0.78-1.04)	0.92 (0.79-1.06)	0.89 (0.77-1.03)	0.301 (w/o 0.170)
Creatinine – 7. DOH, mg/dL (N=169)	0.82 (0.71-0.91)	0.83 (0.73-0.91)	0.81 (0.71-0.92)	0.634 (w/o 0.840)
EGFR - at admission, mL/min (N=215)	90 (77-103)	88 (77-102)	91 (77.25-103) (w/o median 90.16)	0.489 (w/o 0.594)
EGFR – 7. DOH, mL/min (N=169)	101 (89-118)	100.5 (88.5-122.5) (w/o median 101.5)	102 (89-115)	0.815 (w/o 0.854)
Urea - at admission, mg/dL (N=209)	28 (22-36)	29.5 (23.75-37)	26 (21.35)	0.174 (w/o 0.107)
Urea – 7. DOH, mg/dL (N=124)	33 (28-40)	35.5 (28.5-52.5) (w/o median 35.29)	32 (27.25-39.75)	0.170 (w/o 0.164)
ALT - at admission, U/L (N=226)	47 (32.25-68.75)	45 (34-62.5)	48 (32-72.5)	0.506 (w/o 0.574)
ALT – 7. DOH, U/L (N=150)	95 (55-68.75)	118 (55-174)	90 (55-140)	0.224
AST - at admission, U/L (N=219)	48 (35-71.4)	45 (33.5-73)	49.5 (36.78-72.25)	0.142 (w/o 0.155)
AST – 7. DOH, U/L (N=148)	45 (30-69.25)	54 (36-71)	41 (29-64.5)	0.181
GGT - at admission, U/L (N=58)	68 (43-152)	92 (46-120.75)	62 (40-152)	0.972
GGT – 7. DOH, U/L (N=41)	112 (58-192)	160 (57-208)	93.5 (58-160)	0.408
Total bilirubin - at admission, mg/dL (N=165)	0.43 (0.32-0.57)	0.41 (0.32-0.54)	0.44 (0.33-0.58)	0.440
Total bilirubin – 7. DOH, mg/dL (N=63)	0.42 (0.3-0.6)	0.41 (0.28-0.59)	0.43 (0.3-0.6)	0.605
Total calcium – at admission, mmol/L (N=42)	2.14 (2.04-2.23)	2.13 (2.07-2.19)	2.14 (2.04-2.23)	0.959
Total calcium - 7. DOH, mmol/L (N=34)	2.23 (2.1-2.28)	2.18 (2.08-2.28)	2.23 (2.1-2.28)	0.743
Vitamin D3 - at admission, ng/mL (N=81)	26.9 (19.5-33.8)	26.8 (19.75-31.53)	27.7 (19.5-39.2)	0.797 (w/o 0.894)
Vitamin D3 – 7. DOH, ng/mL (N=6)	22.45 (16.7-35.4)	-	22.45 (16.7-35.4)	-

*- Mann-Whitney U test; w/o – without outliers. All variables are presented as median (IQR).

Table S3. Comparison of patients' laboratory parameters (as categorical variables) between the second and the third wave.

Parameter		Total (N=229)	Second wave (N=75)	Third wave (N=154)	p-value
WBC ranges - at admission (N=227)	< 4.5 ×10³/μL	54 (23.79%)	12 (16%)	42 (27.63%)	0.077*
	4.5-10 ×10³/μL	139 (61.23%)	42 (56%)	97 (63.82%)	0.321*
	>10 ×10³/μL	34 (14.98%)	21 (28%)	13 (8.55%)	<0.001*
WBC ranges – 7. DOH (N=187)	< 4.5 ×10³/μL	7 (3.74%)	4 (6.45%)	3 (2.4%)	0.223**
	4.5-10 ×10³/μL	122 (65.24%)	37 (59.68%)	85 (68%)	0.336*
	>10 ×10³/μL	58 (31.02%)	21 (33.87%)	37 (29.6%)	0.670*
Neutrophil count ranges - at admission (N=226)	< 1.9 ×10³/μL	13 (5.75%)	2 (2.67%)	11 (7.28%)	0.229**
	1.9-7 ×10³/μL	160 (70.8%)	45 (60%)	115 (76.16%)	0.018*
	>7 ×10³/μL	53 (23.45%)	28 (37.33%)	25 (16.56%)	0.001*
Neutrophil count ranges - 7. DOH (N=187)	< 1.9 ×10³/μL	5 (2.69%)	3 (4.92%)	2 (1.6%)	0.333**
	1.9-7 ×10³/μL	127 (68.28%)	40 (65.57%)	87 (69.6%)	0.600*
	>7 ×10³/μL	54 (29.03%)	18 (29.51%)	36 (28.8%)	1*
Neutrophil percentage ranges - at admission (N=226)	< 40%	1 (0.44%)	0 (0%)	1 (0.66%)	1**
	40-68%	62 (27.43%)	16 (21.33%)	46 (30.46%)	0.197*
	>68%	163 (72.12%)	59 (78.67%)	104 (68.87%)	0.165*
Neutrophil percentage ranges - 7. DOH (N=186)	< 40%	7 (3.76%)	2 (3.28%)	5 (4%)	1**
	40-68%	113 (60.75%)	39 (63.93%)	74 (59.2%)	0.645*
	>68%	66 (35.48%)	20 (32.79%)	46 (36.8%)	0.709*
Lymphocyte count ranges - at admission (N=226)	< 0.9 ×10³/μL	103 (45.58%)	34 (45.33%)	69 (45.7%)	1*
	0.9-5 ×10³/μL	123 (54.42%)	41 (54.67%)	82 (54.3%)	1*
	>5 ×10³/μL	0	0	0	-
Lymphocyte count ranges - 7. DOH, N(%); (N=186)	< 0.9 ×10³/μL	19 (10.22%)	8 (13.11%)	11 (8.8%)	0.513*
	0.9-5 ×10³/μL	165 (88.71%)	53 (86.89%)	112 (89.6%)	0.762*
	>5 ×10³/μL	2 (1.08%)	0 (0%)	2 (1.6%)	1**
Lymphocyte percentage ranges - at admission (N=226)	< 19%	138 (61.06%)	51 (68%)	87 (57.62%)	0.173*
	19-48%	87 (38.5%)	23 (30.67%)	64 (42.38%)	0.119*
	>48%	1 (0.44%)	1 (1.33%)	0 (0%)	0.332**
Lymphocyte percentage ranges- 7. DOH (N=186)	< 19%	64 (34.41%)	22 (36.07%)	42 (33.6%)	0.867*
	19-48%	118 (63.44%)	37 (60.66%)	81 (64.8%)	0.697*
	>48%	4 (2.15%)	2 (3.28%)	2 (1.6%)	0.599**
PLT ranges- at admission (N=227)	< 150 ×10³/μL	41 (18.06%)	11 (14.67%)	30 (19.74%)	0.453*
	150-400 ×10³/μL	171 (75.33%)	58 (77.33%)	113 (74.34%)	0.743*
	>400 ×10³/μL	15 (6.61%)	6 (8%)	9 (5.92%)	0.757*
PLT ranges – 7. DOH (N=187)	< 150 ×10³/μL	2 (1.07%)	1 (1.61%)	1 (0.8%)	1**
	150-400 ×10³/μL	93 (49.73%)	31 (50%)	62 (49.6%)	1*
	>400 ×10³/μL	92 (49.2%)	30 (48.39%)	62 (49.6%)	0.999*
NLR ≥2 - at admission (N=226)		206 (91.15%)	69 (92%)	137 (90.73%)	0.946*
NLR ≥2- 7. DOH (N=186)		118 (63.44%)	43 (70.49%)	75 (60%)	0.218*
CRP ≥100 mg/L - at admission (N=222)		82 (36.94%)	33 (44%)	49 (33.33%)	0.158*
CRP ≥100 mg/L - 7. DOH (N=187)		9 (4.97%)	6 (10.17%)	3 (2.46%)	0.060**
PCT > 0.5 ng/mL – at admission; (N=205)		22 (10.73%)	10 (14.08%)	12 (8.96%)	0.372*
PCT > 0.5 ng/mL – 7. DOH; (N=102)		10 (9.8%)	5 (14.71%)	5 (7.35%)	0.295**
D-Dimer > 500 μg/L FEU - at admission; (N=206)		154 (74.76%)	47 (83.93%)	107 (71.33%)	0.095*
D-Dimer > 500 μg/L FEU – 7. DOH (N=150)		121 (80.67%)	34 (97.14%)	87 (75.65%)	0.003**
LDH >500 U/L – at admission (N=172)		54 (31.4%)	17 (28.33%)	37 (33.04%)	0.645*
LDH >500 U/L – 7. DOH (N=46)		10 (21.74%)	4 (28.57%)	6 (18.75%)	0.723**

* - Chi-squared with Yates correction; ** - Fisher's exact test. All variables are presented as N (%).

Table S4. Additional data on comparison of general patients' characteristics between survivors and non-survivors.

Parameter		Survivors (N=213)	Non-survivors (N=16)	p-value
BMI, ranges (N=154)	<25	18 (12.95%)	1 (6.67%)	0.696*
	25-29.9	52 (37.41%)	3 (20%)	0.292*
	30-34.9	41 (29.5%)	5 (33.33%)	0.771*
	35-39.9	20 (14.39%)	3 (20%)	0.472*
	≥ 40	8 (5.76%)	3 (20%)	0.077***
Blood type (N=62)	A Rh+	12 (25%)	6 (42.86%)	0.315*
	A Rh-	3(6.25%)	0 (0%)	1***
	B Rh+	11 (22.92%)	1 (7.14%)	0.267*
	B Rh-	2 (4.17%)	2 (14.29%)	0.217***
	AB Rh+	2 (4.17%)	0 (0%)	1***
	AB Rh-	2 (4.17%)	0 (0%)	1***
	0 Rh+	15 (31.25%)	5 (35.71%)	0.755*
	0 Rh-	1 (2.08%)	0 (0%)	1***

* - Chi-squared with Yates correction; ** - Mann-Whitney U test; *** - Fisher's exact test. Continuous variables are presented as median (IQR), categorical variables are presented as N (%).

Table S5. The correlations between patients' general and clinical characteristics (categorical variables) and the need for mechanical ventilation and ICU treatment.

Parameter		Mechanical ventilation				ICU treatment			
		no	yes	phi	p	no	yes	phi	p
Sex (N=229)	female	51 (24.64%)	6 (27.27%)	0.02	0.990*	50 (25.25%)	7 (22.58%)	0.02	0.923*
	male	156 (75.36%)	16 (72.73%)			148 (74.75%)	24 (77.42%)		
BMI, ranges (N=154)	18-24.9 kg/m²	18 (13.53%)	1 (4.76%)	0.09	0.474**	18 (14.17%)	1 (3.7%)	0.12	0.199**
	25-29.9 kg/m²	50 (37.59%)	5 (23.81%)	0.1	0.327**	47 (37.01%)	8 (29.63%)	0.06	0.613*
	30-34.9 kg/m²	40 (30.08%)	6 (28.57%)	0.01	1*	38 (29.92%)	8 (29.63%)	0	1*
	35-39.9 kg/m²	18 (13.53%)	5 (23.81%)	0.1	0.318**	17 (13.39%)	6 (22.22%)	0.09	0.244**
	≥ 40 kg/m²	7 (5.26%)	4 (19.05%)	0.18	0.045**	7 (5.51%)	4 (14.81%)	0.14	0.103**
Comorbidities (N=229)	Comorbidities (any of the following)	79 (38.16%)	14 (63.64%)	0.15	0.037*	76 (38.38%)	17 (54.84%)	0.11	0.124*
	Hypertension	30 (14.49%)	4 (18.18%)	0.03	0.751**	28 (14.14%)	6 (19.35%)	0.05	0.423**
	Asthma	17 (8.21%)	1 (4.55%)	0.04	1**	15 (7.58%)	3 (9.68%)	0.03	0.718**
	Chronic arrhythmia	3 (1.45%)	2 (9.09%)	0.15	0.074**	4 (2.02%)	1 (3.23%)	0.03	0.520**
	Diabetes	13 (6.28%)	5 (22.73%)	0.18	0.019**	11 (5.56%)	7 (22.58%)	0.22	0.005**
	Insulin resistance	4 (1.93%)	2 (.09%)	0.13	0.104**	4 (2.02%)	2 (6.45%)	0.09	0.188**
	Dyslipidemia	5 (2.42%)	1 (4.55%)	0.04	0.458**	5 (2.53%)	1 (3.23%)	0.02	0.587**
	Hypothyroidism	8 (3.86%)	1 (4.55%)	0.01	0.604**	7 (3.54%)	2 (6.45%)	0.05	0.350**
	Hashimoto disease	7 (3.38%)	0 (0%)	0.06	1**	6 (3.03%)	1 (3.23%)	0	1**
Smoking (N=164)	Current	10 (6.58%)	3 (25%)	0.18	0.057**	9 (6.12%)	4 (23.53%)	0.2	0.032**
	Current or former	15 (9.87%)	5 (41.67%)	0.25	0.007**	14 (9.52%)	6 (35.29%)	0.24	0.008**
Symptoms (N=229)	Dyspnea	189 (91.3%)	21 (95.45%)	0.04	1**	181 (91.41)	29 (93.55%)	0.03	1
	Fever	182 (87.92%)	13 (59.09%)	0.24	0.002**	174 (87.88%)	21 (67.74%)	0.19	0.011**
	Cough	180 (86.96%)	15 (68.18%)	0.16	0.028**	173 (87.37%)	22 (70.97%)	0.16	0.027**
	Fatigue	121 (58.45%)	9 (40.91%)	0.1	0.176*	116 (58.59%)	14 (45.16%)	0.09	0.227*
	Diarrhea	40 (19.32%)	1 (4.55%)	0.11	0.139**	40 (20.2%)	1 (3.23%)	0.15	0.041*
	Nausea or vomiting	25 (12.08%)	2 (9.09%)	0.03	1**	26 (13.13%)	1 (3.23%)	0.11	0.140**
	Myalgia	64 (30.92%)	3 (13.64%)	0.11	0.148**	61 (30.81%)	6 (19.35%)	0.09	0.275*
	Sore throat	16 (7.73%)	2 (.09%)	0.01	0.686**	16 (8.08%)	2 (6.45%)	0.02	1**
	Headache	41 (19.81%)	1 (4.55%)	0.12	0.088**	39 (19.7%)	3 (9.68%)	0.09	0.275*
	Smell and/or taste disorders	40 (19.32%)	3 (13.64%)	0.04	0.774**	38 (19.19%)	5 (16.13%)	0.03	0.874*
	Smell disorders	34 (16.43%)	3 (13.64%)	0.02	1**	32 (16.16%)	5 (16.13%)	0	1*
	Taste disorders	34 (16.43%)	3 (13.64%)	0.02	1**	32 (16.16%)	5 (16.13%)	0	1*
	Hemoptysis	7 (3.38%)	1 (4.55%)	0.02	0.560**	6 (3.03%)	2 (6.45%)	0.06	0.296*
Lung involvement on CT ≥ 50% (%) (N=207)		38 (20.11%)	13 (72.22%)	0.34	<0.001**	35 (19.13%)	16 (66.67%)	0.35	<0.001*
SpO2 at admission <90% (N=190)		77 (44%)	11 (73.33%)	0.16	0.055*	74 (43.53%)	14 (70%)	0.16	0.045*
Conventional oxygen therapy (N=229)		200 (96.62%)	22 (100%)	0.06	1**	191 (96.46%)	31 (100%)	0.07	0.598**
HFNO (N=229)		36 (17.39%)	19 (86.36%)	0.48	<0.001*	29(14.65%)	26 (83.87%)	0.55	<0.001*
Mechanical ventilation (N=229)		-	-	-	-	1 (0.51%)	21 (67.74%)	0.78	<0.001**
ECMO (N=229)		5 (2.42%)	3 (13.64%)	0.18	0.032**	6 (3.03%)	2 (6.45%)	0.06	0.296**
ICU admission (N=229)		10 (4.83%)	21 (95.45%)	0.78	<0.001**	-	-	-	-
Vasopressors (N=229)		0 (0%)	21 (95.45%)	0.97	<0.001**	1 (0.51%)	20 (64.52%)	0.76	<0.001**
CRRT (N=229)		0 (0%)	5 (22.73%)	0.46	<0.001**	0 (0%)	5 (16.13%)	0.38	<0.001**

* chi-squared test with Yates' correction; ** Fisher's exact test; All variables are presented as N (%).

Table S6. The correlations between patients' general and clinical characteristics and laboratory parameters (presented as continuous variables) and the need for mechanical ventilation and ICU treatment.

Parameter	Mechanical ventilation		ICU treatment	
	rho	p-value*	rho	p-value*
Age (N=229)	0.12	0.066	0.04	0.592
Weight (N=160)	0.19 (w/o 0.17)	0.016 (w/o 0.033)	0.14 (w/o 0.12)	0.076 (w/o 0.120)
BMI (N=154)	0.21 (w/o 0.17)	0.009 (w/o 0.040)	0.19 (w/o 0.16)	0.017 (w/o 0.054)
Percentage of lung involvement on CT (N=207)	0.35	<0.001	0.36	<0.001
SpO2 at admission (N=190)	0.25 (w/o 0.15)	<0.001 (w/o 0.034)	-0.2 (w/o 0.16)	<0.001 (w/o 0.026)
WBC - at admission (N=227)	0.26	<0.001	0.28	<0.001
WBC - 7. DOH (N=187)	0.32 (w/o 0.28)	<0.001	0.25 (w/o 0.22)	<0.001 (w/o 0.003)
Neutrophil count - at admission (N=226)	0.27	<0.001	0.3	<0.001
Neutrophil count - 7. DOH (N=186)	0.39 (w/o 0.38)	<0.001	0.36	<0.001
Neutrophil percentage - at admission (N=226)	0.2	0.002 (w/o 0.003)	0.27	<0.001
Neutrophil percentage - 7. DOH (N=186)	0.34	<0.001	0.39	<0.001
Lymphocyte count - at admission (N=226)	0.01 (w/o 0.02)	0.898 (w/o 0.821)	0.08 (w/o 0.07)	0.237 (w/o 0.276)
Lymphocyte count - 7. DOH (N=186)	-0.25	<0.001 (w/o 0.001)	-0.33	<0.001
Lymphocyte percentage - at admission (N=226)	-0.24 (w/o 0.23)	<0.001	-0.31	<0.001
Lymphocyte percentage - 7. DOH (N=186)	-0.43	<0.001	-0.46	<0.001
NLR - at admission (N=226)	0.23	<0.001 (w/o 0.001)	0.3 (w/o 0.31)	<0.001
NLR - 7. DOH (N=186)	0.42 (w/o 0.39)	<0.001	0.45 (w/o 0.43)	<0.001
IG count - at admission (N=217)	0.32 (w/o 0.30)	<0.001	0.35 (w/o 0.32)	<0.001
IG count - 7. DOH (N=186)	0.29 (w/o 0.21)	<0.001 (w/o 0.005)	0.20 (w/o 0.13)	0.007 (w/o 0.068)
IG percentage – at admission (N=217)	0.33 (w/o 0.29)	<0.001	0.37 (w/o 0.33)	<0.001
IG percentage – 7. DOH (N=186)	0.24 (w/o 0.18)	<0.001 (w/o 0.016)	0.16 (w/o 0.11)	0.028 (w/o 0.137)
RBC count - at admission (N=227)	0.06	0.363 (w/o 0.400)	0.05	0.420 (w/o 0.473)
RBC count - 7. DOH (N=187)	-0.41 (w/o -0.42)	<0.001	-0.34	<0.001
hemoglobin - at admission to hospital (N=227)	0.01	0.830 (w/o 0.923)	-0.01 (w/o -0.02)	0.929 (w/o 0.818)
hemoglobin - 7. DOH (N=187)	-0.42	<0.001	-0.33	<0.001
hematocrit - at admission (N=227)	0.05	0.435 (w/o 0.472)	0.04	0.537 (w/o 0.592)
hematocrit - 7. DOH (N=187)	-0.36 (w/o -0.37)	<0.001	-0.29 (w/o -0.30)	<0.001
PLT - at admission to hospital (N=227)	0.14 (w/o 0.15)	0.038 (w/o 0.025)	0.19 (w/o 0.17)	0.003 (w/o 0.012)
PLT - 7. DOH (N=187)	-0.04 (w/o -0.01)	0.573 (w/o 0.911)	-0.03 (w/o 0)	0.683 (w/o 0.975)
CRP - at admission (N=222)	0.29 (w/o 0.3)	<0.001	0.28 (w/o 0.27)	<0.001
CRP - 7. DOH (N=181)	0.48 (w/o 0.43)	<0.001	0.51 (w/o 0.48)	<0.001
Ferritin - at admission (N=61)	0.29 (w/o 0.3)	0.025 (w/o 0.018)	0.22 (w/o 0.24)	0.093 (w/o 0.068)
Ferritin - 7. DOH (N=31)	0.12	0.529	0.38	0.037
IL-6 - at admission (N=116)	0.18	0.049	0.11	0.236
IL-6 - 7. DOH (N=53)	0.33	0.015	0.43	0.001
AT III - at admission (N=17)	0.16	0.542	0.12	0.650
AT III - 7. DOH (N=10)	-0.29	0.413	-0.29	0.413
PCT - at admission (N=205)	0.38 (w/o 0.039)	<0.001	0.37 (w/o 0.39)	<0.001
PCT - 7. DOH (N=102)	0.56	<0.001	0.61	<0.001
D-Dimer - at admission (N=206)	0.24	<0.001 (w/o 0.001)	0.27	<0.001
D-Dimer - 7. DOH (N=150)	0.36	<0.001	0.36	<0.001
PT - at admission (N=198)	0.03 (w/o -0.02)	0.672 (w/o 0.812)	0.12 (w/o 0.11)	0.093 (w/o 0.126)
PT - 7. DOH (N=80)	0.17 (w/o 0.18)	0.139 (w/o 0.113)	0.26 (w/o 0.28)	0.020 (w/o 0.013)
INR - at admission (N=199)	0.02 (w/o -0.03)	0.768 (w/o 0.780)	0.12 (w/o 0.07)	0.092 (w/o 0.469)
INR - 7. DOH (N=80)	0.15 (w/o 0.16)	0.183 (w/o 0.150)	0.25 (w/o 0.26)	0.028 (w/o 0.109)
APTT - at admission (N=196)	-0.13 (w/o -0.12)	0.077 (w/o 0.101)	-0.12 (w/o -0.11)	0.091 (w/o 0.128)
APTT - 7. DOH (N=65)	0.09	0.454	0.17	0.167
Fibrinogen - at admission (N=62)	-0.08 (w/o 0.04)	0.538 (w/o 0.751)	-0.07 (w/o -0.04)	0.574 (w/o 0.740)
Fibrinogen - 7. DOH (N=39)	0.15	0.355	0.14	0.402
LDH - at admission (N=172)	0.37	<0.001	0.35	<0.001

LDH - 7. DOH (N=46)	0.36	0.014	0.41	0.005
hs-TnI - at admission (N=153)	0.39	<0.001	0.33	<0.001
hs-TnI - 7. DOH (N=40)	0.55	<0.001	0.56	<0.001
CK - at admission (N=142)	0.14	0.088	0.17	0.046
CK - 7. DOH (N=38)	0.55	<0.001	0.43	0.007
CK-MB - at admission (N=124)	0.22	0.014	0.21	0.017
CK-MB - 7. DOH (N=27)	0.35	0.074	0.26	0.186
NT-proBNP - at admission (N=142)	0.29	<0.001	0.32	<0.001
NT-proBNP - 7. DOH (N=42)	0.48	0.001	0.39	0.010
Myoglobin - at admission (N=15)	0.69	0.004	0.39	0.147
Myoglobin - 7. DOH (N=11)	0.60	0.053	0.60	0.053
Creatinine - at admission (N=222)	0.22 (w/o 0.18)	0.001 (w/o 0.006)	0.11 (w/o 0.11)	0.090 (w/o 0.114)
Creatinine - 7. DOH (N=169)	0.03 (w/o -0.05)	0.695 (w/o 0.485)	0 (w/o -0.07)	0.959 (w/o 0.355)
EGFR - at admission to hospital (N=215)	-0.24	<0.001	-0.1	0.132
EGFR - 7. DOH (N=169)	-0.02 (w/o 0.05)	0.835 (w/o 0.488)	0.04 (w/o 0.1)	0.598 (w/o 0.204)
Urea - at admission (N=209)	0.28 (w/o 0.24)	<0.001	0.26 (w/o 0.25)	<0.001
Urea - 7. DOH (N=124)	0.53 (w/o 0.47)	<0.001	0.56 (w/o 0.52)	<0.001
ALT - at admission (N=226)	0 (w/o -0.01)	0.978 (w/o 0.836)	0.01 (w/o 0.02)	0.839 (w/o 0.779)
ALT - 7. DOH (N=150)	-0.3	<0.001	-0.24	0.003
AST - at admission (N=219)	0.13	0.057	0.09	0.203
AST - 7. DOH (N=148)	-0.13	0.124	-0.07	0.365
GGT - at admission (N=58)	0.24	0.064	0.21	0.113
GGT - 7. DOH (N=41)	-0.03	0.858	0.01	0.931
Total bilirubin - at admission (N=165)	0.13	0.089	0.16	0.039
Total bilirubin - 7. DOH (N=63)	-0.20	0.113	-0.06	0.625
Albumin – at admission (N=26)	-0.42	0.032	-0.44	0.025
Total calcium – at admission (N=42)	-0.41	0.007	-0.33	0.036
Total calcium - 7. DOH (N=34)	-0.51	0.002	-0.49	0.004
Vitamin D3 - at admission (N=81)	-0.08 (w/o -0.13)	0.502 (w/o 0.250)	-0.02 (w/o -0.07)	0.887 (w/o 0.559)
Vitamin D3 - 7. DOH (N=6)	-	-	-0.83	0.042
Time from the onset of symptoms to hospital admission (N=229)	-0.14	0.034	-0.10	0.441
Maximum flow - HFNO (N=55)	0.27	0.046	0.42	0.002
Maximum FiO2 – HFNO (N=55)	0.29	0.030	0.32	0.019
Time from the onset of symptoms to ICU admission (N=31)	-0.21	0.257	-	-

w/o – without outliers. * - Spearman's correlation.

Table S7. The correlations between patients' laboratory parameters (presented as categorical variables) and the need for mechanical ventilation and ICU treatment.

Parameter		Mechanical ventilation				ICU treatment			
		no	yes	phi	p	no	yes	phi	p
WBC - at admission, ranges (N=227)	<4.5 ×10^3/μL	51 (24.88%)	3 (13.64%)	0.08	0.361*	50 (25.1%)	4 (12.9%)	0.1	0.192*
	4.5-10 ×10^3/μL	132 (64.39%)	7 (31.82%)	0.2	0.006*	125 (63.78%)	14 (45.16%)	0.13	0.075*
	>10 ×10^3/μL	22 (10.73%)	12 (54.55%)	0.36	<0.001**	21 (10.71%)	13 (41.94%)	0.3	<0.001**
WBC – 7. DOH, ranges (N=187)	<4.5 ×10^3/μL	7 (4.17%)	0 (0%)	0.07	1**	7 (4.43%)	0 (0%)	0.08	0.598**
	4.5-10 ×10^3/μL	116 (69.05%)	6 (31.58%)	0.24	0.003*	109 (68.99%)	13 (44.83%)	0.18	0.021*
	>10 ×10^3/μL	45 (26.79%)	13 (68.42%)	0.27	0.001*	42 (26.58%)	16 (55.17%)	0.22	0.004*
Neutrophil count - at admission, ranges (N=226)	<1.9 ×10^3/μL	13 (6.37%)	0 (0%)	0.08	0.622**	13 (6.67%)	0 (0%)	0.1	0.224**
	1.9-7 ×10^3/μL	152 (74.51%)	8 (36.36%)	0.25	<0.001*	148 (75.9%)	12 (38.71%)	0.28	<0.001*
	>7 ×10^3/μL	39 (19.12%)	14 (63.64%)	0.31	<0.001*	34 (17.44%)	19 (61.29%)	0.36	<0.001*
Neutrophil count - 7. DOH, ranges (N=186)	<1.9 ×10^3/μL	5 (2.99%)	0 (0%)	0.06	1**	5 (3.18%)	0 (0%)	0.07	1**
	1.9-7 ×10^3/μL	124 (74.25%)	3 (15.79%)	0.38	<0.001*	117 (74.53%)	10 (34.48%)	0.31	<0.001*
	>7 ×10^3/μL	38 (22.75%)	16 (84.21%)	0.41	<0.001*	35 (22.29%)	19 (65.52%)	0.35	<0.001*
Neutrophil percentage - at admission, ranges (N=226)	<40%	1 (0.49%)	0 (0%)	0.02	1**	1 (0.51%)	0 (0%)	0.03	1**
	40-68%	62 (30.39%)	0 (0%)	0.2	0.005*	61 (31.28%)	1 (3.23%)	0.22	0.002*
	>68%	141 (69.12%)	22 (100%)	0.2	0.005*	133 (68.21%)	30 (96.77%)	0.22	0.002*
Neutrophil percentage - 7. DOH, ranges (N=186)	<40%	7 (4.19%)	0 (0%)	0.07	1**	7 (4.46%)	0 (0%)	0.08	0.598**
	40-68%	108 (64.67%)	5 (26.32%)	0.24	0.003*	105 (66.88%)	8 (27.59%)	0.29	<0.001*
	>68%	52 (31.14%)	14 (73.68%)	0.27	0.001*	45 (28.66%)	21 (72.41%)	0.33	<0.001*
Lymphocyte count - at admission, ranges (N=226)	<0.9 ×10^3/μL	94 (46.08%)	9 (40.91%)	0.03	0.812*	87 (44.62%0	16 (51.61%)	0.05	0.594*
	0.9-5 ×10^3/μL	110 (53.92%)	13 (59.09%)	0.03	0.812*	108 (55.38%)	15 (48.39%)	-0.05	0.594*
	>5 ×10^3/μL	0 (0%)	0 (0%)	-	-	0 (0%)	0 (0%)	-	-
Lymphocyte count - 7. DOH, ranges (N=186)	<0.9 ×10^3/μL	12 (7.19%)	7 (36.84%)	0.2	0.001**	11 (7.01%)	8 (27.59%)	0.25	0.038**
	0.9-5 ×10^3/μL	153 (91.62%)	12 (63.16%)	0.27	0.002**	144 (91.72%)	21 (72.41%)	0.22	0.007**
	>5 ×10^3/μL	2 (1.2%)	0 (0%)	0.04	1**	2 (1.27%)	0 (0%)	0.04	1**
Lymphocyte percentage - at admission, ranges (N=226)	<19%	116 (56.86%)	22 (100%)	0.26	<0.001*	108 (55.38%)	30 (96.77%)	0.29	<0.001*
	19-48%	87 (42.65%)	0 (0%)	0.26	<0.001*	86 (44.1%)	1 (3.23%)	0.29	<0.001*
	>48%	1 (0.49%)	0 (0%)	0.02	1**	1 (0.51%)	0 (0%)	0.03	1**
Lymphocyte percentage - 7. DOH, ranges (N=186)	<19%	47 (28.14%)	17 (89.47%)	0.39	<0.001*	40 (25.48%)	24 (82.76%)	0.44	<0.001*
	19-48%	116 (69.46%)	2 (10.53%)	0.37	<0.001*	113 (71.97%)	5 (17.24%)	0.41	<0.001*
	>48%	4 (2.4%)	0 (0%)	0.05	1**	4 (2.55%)	0 (0%)	0.06	1**
NLR ≥2 - at admission (N=226)		184 (90.2%)	22 (100%)	0.1	0.231**	176 (90.26%)	30 (96.77%)	0.08	0.324**
NLR ≥2- 7. DOH (N=186)		99 (59.28%)	19 (100%)	0.26	0.001*	90 (57.32%)	28 (96.55%)	0.30	<0.001*
PLT - at admission, ranges (N=227)	<150 ×10^3/μL	40 (19.51%)	1 (4.55%)	0.12	0.139**	41 (20.92%)	0 (0%)	0.19	0.010*
	150-400 ×10^3/μL	155 (75.61%)	16 (72.73%)	0.02	0.970*	147 (75%)	24(77.42%)	0.02	0.947*
	>400 ×10^3/μL	10 (4.88%)	5 (22.73%)	0.21	0.008**	8 (4.08%)	7 (22.58%)	0.26	0.001**
PLT – 7. DOH, ranges (N=187)	<150 ×10^3/μL	1 (0.6%)	1 (5.26%)	0.14	0.193**	1 (0.63%)	1 (3.45%)	0.1	0.287**
	150-400 ×10^3/μL	85 (50.6%)	8 (42.11%)	0.05	0.646*	80 (50.63%)	13 (44.83%)	0.04	0.709*
	>400 ×10^3/μL	82 (48.81%)	10 (52.63%)	0.02	0.941*	77 (48.73%)	15 (51.72%)	0.02	0.925*
CRP>100 mg/L – at admission (N=222)		66 (32.84%)	16 (76.19%)	0.26	<0.001	61 (31.94%)	21 (67.75%)	0.26	<0.001
CRP>100 mg/L – 7. DOH (N=181)		2 (1.24%)	7 (35%)	0.49	<0.001	2 (1.32%)	7 (24.14%)	0.39	<0.001
PCT > 0.5 ng/mL – at admission (N=205)		12 (6.56%)	10 (45.45%)	0.39	<0.001**	11 (6.32%)	11 (35.48%)	0.34	<0.001**
PCT > 0.5 ng/mL – 7. DOH (N=102)		3 (3.57%)	7 (38.89%)	0.45	<0.001**	2 (2.63%)	8 (30.77%)	0.41	<0.001**
D-Dimer > 500 μg/L FEU - at admission; (N=206)		133 (71.89%)	21 (100%)	0.20	0.003**	125 (71.02%)	29 (96.67%)	0.21	0.001**
D-Dimer > 500 μg/L FEU – 7. DOH; (N=150)		101 (77.69%)	20 (13.33%)	0.19	0.014**	93 (76.23%)	28 (100%)	0.23	0.002**
LDH >500 U/L – at admission (N=172)		41 (26.11%)	13 (86.67%)	0.37	<0.001**	37 (24.5%)	17 (80.95%)	0.40	<0.001**
LDH >500 U/L – 7. DOH (N=46)		8 (19.05%)	2 (50%)	0.21	0.201**	6 (15.79%)	4 (50%)	0.31	0.055**

* chi-squared test with Yates' correction; ** Fisher's exact test; All variables are presented as N (%).

Table S8. Univariate logistic regression analysis of COVID-19 symptoms for the prediction of death, mechanical ventilation and ICU treatment.

Symptom	death			MV			ICU treatment		
	OR	95%CI	p	OR	95%CI	p	OR	95%CI	p
Dyspnoea	1.39	0.17-11.1	0.202	2	0.25-15.75	0.510	1.36	0.3-6.21	0.690
Fever	0.25	0.09-0.75	0.013	0.2	0.08-0.51	<0.001	0.29	0.12-0.69	0.005
Cough	0.25	0.09-0.75	0.013	0.32	0.12-0.86	0.024	0.35	0.15-0.85	0.021
Fatigue	0.57	0.2-1.59	0.281	0.49	0.2-1.2	0.120	0.58	0.27-1.25	0.164
Dairrhoea	0.29	0.04-2.25	0.235	0.2	0.03-1.53	0.200	0.13	0.02-1	0.049
Nausea or vomiting	0.48	0.06-3.78	0.486	0.73	0.16-3.3	0.681	0.22	0.03-1.69	0.145
Myalgia	0.33	0.07-1.47	0.145	0.35	0.1-1.24	0.103	0.54	0.21-1.38	0.198
Sore throat	0.77	0.1-6.18	0.805	1.19	0.26-5.57	0.822	0.78	0.17-3.59	0.755
Headache	0.28	0.04-2.18	0.224	0.19	0.03-1.48	0.113	0.44	0.13-1.51	0.191
Smell or taste disorders	0.6	0.13-2.74	0.509	0.66	0.19-2.34	0.519	1	0.36-2.79	0.996
Smell disorders	0.72	0.16-3.34	0.681	0.8	0.23-2.87	0.736	1	0.36-2.79	0.996
Taste disorders	0.72	0.16-3.34	0.681	0.8	0.23-2.87	0.736	0.81	0.29-2.25	0.685
